# Identification and characterization of a prokaryotic 6-4 photolyase from *Synechococcus elongatus* with a deazariboflavin antenna chromophore

**DOI:** 10.1093/nar/gkac416

**Published:** 2022-05-27

**Authors:** Simeng Chen, Chenxi Liu, Chenchen Zhou, Zhihui Wei, Yuting Li, Lei Xiong, Liang Yan, Jun Lv, Liang Shen, Lei Xu

**Affiliations:** Anhui Province Key Laboratory of Active Biological Macro-molecules, Wannan Medical College, Wuhu, Anhui 241002, China; Anhui Province Key Laboratory of Active Biological Macro-molecules, Wannan Medical College, Wuhu, Anhui 241002, China; Anhui Province Key Laboratory of Active Biological Macro-molecules, Wannan Medical College, Wuhu, Anhui 241002, China; Anhui Province Key Laboratory of Active Biological Macro-molecules, Wannan Medical College, Wuhu, Anhui 241002, China; Anhui Province Key Laboratory of Active Biological Macro-molecules, Wannan Medical College, Wuhu, Anhui 241002, China; Anhui Province Key Laboratory of Active Biological Macro-molecules, Wannan Medical College, Wuhu, Anhui 241002, China; Anhui Province Key Laboratory of Active Biological Macro-molecules, Wannan Medical College, Wuhu, Anhui 241002, China; Anhui Province Key Laboratory of Active Biological Macro-molecules, Wannan Medical College, Wuhu, Anhui 241002, China; College of Life Sciences, Anhui Normal University, Wuhu, Anhui 241000, China; Anhui Province Key Laboratory of Active Biological Macro-molecules, Wannan Medical College, Wuhu, Anhui 241002, China

## Abstract

*Synechococcus elongatus*, formerly known as *Anacystis nidulans*, is a representative species of cyanobacteria. It is also a model organism for the study of photoreactivation, which can be fully photoreactivated even after receiving high UV doses. However, for a long time, only one photolyase was found in *S. elongatus* that is only able to photorepair UV induced cyclobutane pyrimidine dimers (CPDs) in DNA. Here, we characterize another photolyase in *S. elongatus*, which belongs to iron-sulfur bacterial cryptochromes and photolyases (FeS-BCP), a subtype of prokaryotic 6–4 photolyases. This photolyase was named *Se*PhrB that could efficiently photorepair 6–4 photoproducts in DNA. Chemical analyses revealed that *Se*PhrB contains a catalytic FAD cofactor and an iron-sulfur cluster. All of previously reported FeS-BCPs contain 6,7-dimethyl-8-ribityllumazine (DMRL) as their antenna chromophores. Here, we first demonstrated that *Se*PhrB possesses 7,8-didemethyl-8-hydroxy-5-deazariboflavin (8-HDF) as an antenna chromophore. Nevertheless, *Se*PhrB could be photoreduced without external electron donors. After being photoreduced, the reduced FAD cofactor in *Se*PhrB was extremely stable against air oxidation. These results suggest that FeS-BCPs are more diverse than expected which deserve further investigation.

## INTRODUCTION

Cyanobacteria are among the oldest living organisms on the earth, which began to perform oxygenic photosynthesis ∼3 billion years ago (1,2). They also contribute a substantial fraction of global primary production (3). Cyanobacteria do these works relying on sunlight as the energy source. Meanwhile, they suffer from the UV component of sunlight, which induces cyclobutane pyrimidine dimer (CPD, ∼70–90%) and the (6–4) pyrimidine-pyrimidone photoproduct (6–4 photoproduct, ∼10–30%) in DNA (4,5). These harmful lesions must be repaired to restore the normal functions of DNA. Indeed, it is well known that cyanobacteria possess an efficient DNA repair mechanism, named photoreactivation, through which the UV-induced lesions were directly repaired by enzymes called photolyases (EC 4.1.99.3) using external light energy (6,7).

*Synechococcus elongatus* is a famous representative species of cyanobacteria, which was known as *Anacystis nidulans* for many years (8). It is a model organism for the studies of transformational competence (9), circadian clock (10), as well as photoreactivation (6,11). It was shown that *S. elongatus* could be fully recovered by photoreactivation after receiving extensive UV irradiation (11). A CPD photolyase gene of *S. elongatus* was cloned and heterologously expressed in *Escherichia coli* cells (12–14). Compared with the photolyase purified from the *S. elongatus* cells (7), *E. coli* expressed *S. elongatus* CPD photolyase is still active that contains a catalytic cofactor FAD, but lacks an antenna cofactor 7,8-didemethyl-8-hydroxy-5-deazariboflavin (8-HDF, or named FO) (13,14). The structure of *S. elongatus* CPD photolyase was well resolved (15), and the first photolyase-CPD-like lesion complex structure was obtained using this enzyme (16). However, a discrepancy existed that only one photolyase found in *S. elongatus* could not be responsible for the full recovery of photoreactivation, because the 6–4 photoproducts was not repaired by the CPD photolyase.

A novel type of photolyases named iron-sulfur bacterial cryptochromes and photolyases (FeS-BCP) was discovered in *Agrobacterium fabrum* (formerly known as *Agrobacterium tumefaciens*) (17,18), *Rhodobacter sphaeroides* (19,20), *Vibrio cholerae* (21), and *Sphingomonas* sp. (22). And it was proposed that FeS-BCPs should be broadly distributed among prokaryotes (18). Biochemical and structural studies revealed that these FeS-BCPs can photorepair 6–4 photoproducts, and have a catalytic FAD cofactor, an iron-sulfur cluster, and a unique antenna cofactor 6,7-dimethyl-8-ribityllumazine (DMRL) (18–22). It was also shown that *R. sphaeroides* FeS-BCP (*Rs*CryB) has bacterial cryptochrome functions that regulates photosynthesis and energy metabolism gene expression (23). Nevertheless, it was recently found that some other prokaryotic 6–4 photolyases containing no iron-sulfur cluster, such as *Prochlorococcus marinus* PromaPL, were classified into the same phylogenetic group together with FeS-BCPs (24). Therefore, it is better to name the entire group ‘prokaryotic 6–4 photolyases’, and to regard FeS-BCPs as a subtype of it.

Genome sequence analysis showed that *S. elongatus* contains three photolyase/cryptochrome genes: a previously described CPD photolyase gene (*SephrA*), a FeS-BCP gene (*SephrB*), and a short photolyase-like (SPL) gene (*SephrC*) (25). In this study, we cloned the *SephrB* gene and expressed it in *Escherichia coli* cells. As expected, *Se*PhrB has a catalytic FAD cofactor and an iron–sulfur cluster with 6–4 photolyase activity. However, no antenna cofactor was detected in *E. coli* expressed *Se*PhrB. Heterologously expressing a FO synthase enables biosynthesis of 8-HDF *in E. coli* (26,27). When *Se*PhrB and the FO synthase of *Streptomyces coelicolor* were co-expressed *in E. coli*, the isolated *Se*PhrB protein carried an additional cofactor 8-HDF, which had also increased repair activity. To our knowledge, this is a first report that a FeS-BCP possesses 8-HDF as its antenna cofactor instead of DMRL. Nevertheless, it was found that *Se*PhrB could be photoreduced in the absence of an external electron donor, and photoreduced *Se*PhrB was extremely stable against air oxidation. The unique properties of *Se*PhrB implies that FeS-BCPs are more diverse than expected, which need further investigation to better understand this type of proteins.

## MATERIALS AND METHODS

### Sequence analyses and structure prediction

**Figure 1.**
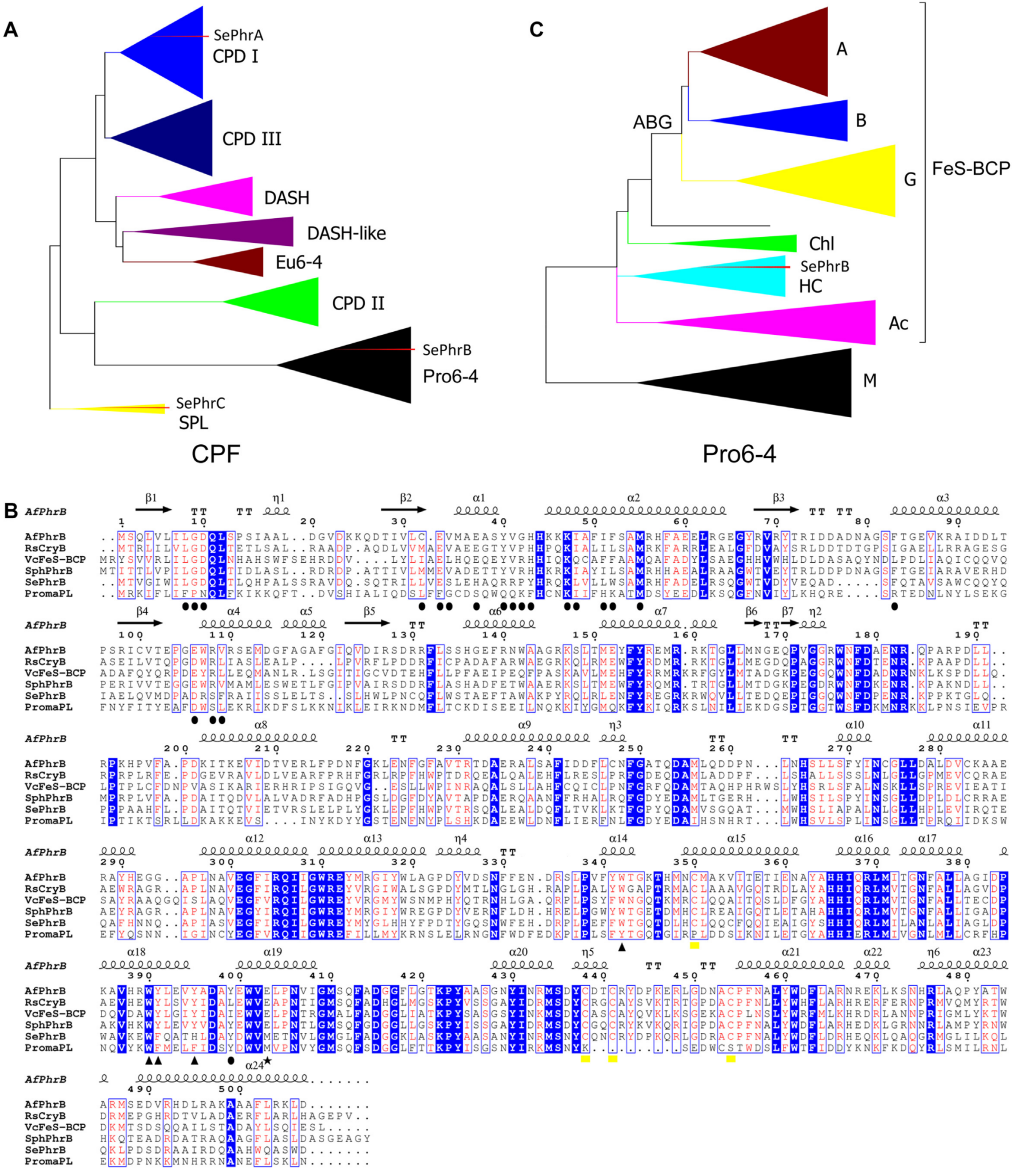
Sequence analyses of *Se*PhrB and other cryptochrome/photolyase family (CPF) proteins. (**A**) Phylogenetic tree of 574 CPF proteins from 271 organisms of all life kingdoms generated by the maximum likelihood method. Eight main groups were obtained: class I CPD photolyases (CPD I), class III CPD photolyases (CPD III), DASH proteins (DASH), DASH-like proteins (DASH-like), eukaryotic 6–4 photolyases (Eu6-4), class II CPD photolyases (CPD II), prokaryotic 6–4 photolyases (Pro6-4), and Short Photolyase-Likes (SPL). The positions of the three *S. elongatus* CPF proteins *Se*PhrA, *Se*PhrB, and *Se*PhrC are shown by red arrow heads. (**B**) Phylogenetic tree of 110 prokaryotic 6–4 photolyases generated by the maximum likelihood method. Seven groups were obtained: A, B, G, Chl, HC, Ac and M. The subgroups A, B, and G constitute a large cluster ABG. The members except for those in the subgroup M belong to a subtype called iron-sulfur bacterial cryptochromes and photolyases (FeS-BCPs). The position of *Se*PhrB is shown by a red arrow head. The details of the tree are shown in Supplementary Figure S1. (**C**) Sequence alignment of *Se*PhrB with *A. fabrum* FeS-BCP (*Af*PhrB), *R. sphaeroides* FeS-BCP (*Rs*CryB), *V. cholerae* FeS-BCP (*Vc*FeS-BCP), *Sphingomonas* sp. FeS-BCP (*Sph*PhrB), and *P. marinus* PromaPL. The putative antenna cofactor binding sites are marked with circles. The sites of the putative electron transfer chain are marked with circles triangles. The site that near the N5 position of the FAD cofactor is marked with a star. The iron-sulfur cluster coordination sites are marked with yellow boxes.

To find out the photolyase/cryptochrome genes in the genome of *S. elongatus* PCC 7942, TBLASTN searches were performed on the web site (http://blast.ncbi.nlm.nih.gov) using three query protein sequences: *E. coli* class I CPD photolyase (*Ec*CPDI, accession number: 1DNP_A), *Methanosarcina mazei* class II CPD photolyase (*Mm*CPDII, 2XRY_A), and *A. fabrum* FeS-BCP (*Af*PhrB, 4DJA_A). The whole genome shotgun sequences of *S. elongatus* PCC 7942 (NCBI assembly: GCF_014698905.1) (28) was set as the search database. The query of *Ec*CPDI got two hits, H6G84_07260 and H6G84_03645; the query of *Mm*CPDII got one hit, H6G84_07260; and the query of *Af*PhrB got two hits, H6G84_09140 and H6G84_03645. The same hits obtained by different types of queries implied that the hit genes might be evolutionary intermediates. To clarify the exact types of the proteins encoded by the hit genes, phylogenetic analysis was performed by MEGA 7.0 (29) with the three protein sequences and the other 571 sequences from 270 organisms of all life kingdoms retrieved by the same method. The sequences were divided into eight main groups: class I CPD photolyases, class III CPD photolyases, DASHs, DASH-likes, eukaryotic 6–4 photolyases, class II CPD photolyases, prokaryotic 6–4 photolyases (including FeS-BCPs), and SPLs (Figure 1A) (25). The H6G84_07260, H6G84_09140 and H6G84_03645 encoding proteins were distributed into the class I CPD photolyase group, the prokaryotic 6–4 photolyase group, and the SPL group, respectively. We named these genes *SephrA*, *SephrB* and *SephrC* in order. And their encoding proteins were named *Se*PhrA, *Se*PhrB and *Se*PhrC, respectively.

To get further information of the category of prokaryotic 6–4 photolyases, *Af*PhrB was used as the query, and TBLASTN searches were performed as above. A total of 110 prokaryotic 6–4 photolyase sequences were retrieved. Phylogenetic analysis was performed by MEGA 7.0 using the maximum likelihood method and 500 bootstrap iterations.

To explore the primary structural of prokaryotic 6–4 photolyases, six representatives including *Af*PhrB (17,18), *Rs*CryB (19,20), *V. cholerae* FeS-BCP (*Vc*FeS-BCP) (21), *Sphingomonas* sp. FeS-BCP (*Sph*PhrB) (22), *P. marinus* PromaPL (24), and *Se*PhrB were selected and aligned by using Clustal W (30). The result of the alignment was rendered by using ESPript 3.0 (http://espript.ibcp.fr/ESPript/ESPript/) (31). The putative antenna cofactor binding, the electron transfer chain, and iron-sulfur cluster coordination sites were assigned based on the structural information of *Af*PhrB and *Rs*CryB (18,19). And the conservation of these sites was analyzed using WebLogo (32). The protein structure of *Se*PhrB was predicted using AlphaFold2 with default options (33). Five models were obtained and the best one with a predicted local-distance difference test (pLDDT) score of 95.86 was used for structural analyses.

### Strains, gene cloning, protein expression and purification

The algal strain *S. elongatus* PCC 7942 was purchased from the Freshwater Algae Culture Collection at the Institute of Hydrobiology, Wuhan, China. The *SephrA* and *SephrB* genes were amplified from the genomic DNA of *S. elongatus* PCC 7942, and cloned into the pET22b expression vector (Novagen) to obtain pETSePhrA and pETSePhrB plasmids, respectively. The *A. fabrum phrB* gene was chemically synthesized (General Biol Inc.), and cloned into the pET22b to obtain pETAfPhrB plasmid. The *E. coli* BL21(DE3) cells transformed with the recombinant plasmid were grown at 37°C in LB medium containing 50 μg ml^–1^ ampicillin until *A*_600 nm_ reached 0.6–0.8. Then protein overexpression was induced with 0.5 mM isopropyl-β-d-thiogalactopyranoside (IPTG). The cells were further incubated at 20°C for 20 h, then collected by centrifugation, resuspended in Start buffer (50 mM Tris–HCl, pH 7.2, 200 mM NaCl, 10 mM imidazole and 10% glycerol), and disrupted by sonication. The supernatant of the lysate was loaded onto a Ni-NTA Sefinose Resin 6FF column (BBI), and washed with Start buffer. The target protein was eluted from the column using Elution buffer (50 mM Tris–HCl, pH 7.2, 200 mM NaCl, 250 mM imidazole and 10% glycerol), and further purified using a Superdex 200 increase 10/300 GL column (GE Healthcare). The purified protein was stored in Protein buffer containing 50 mM Tris–HCl, pH 7.2, 200 mM NaCl and 10% glycerol.

To co-express proteins and 8-HDF in *E. coli*, a method adapted from a previous study (27) was used. The *S. coelicolor fbiC* gene was obtained by chemical synthesis with its codons being optimized for *E. coli* expression (General Biol Inc.), and cloned into the pCDFDuet-1 vector (Novagen), to obtain pCDFScFbiC plasmid. The pCDFDuet-1 vector and the derived plasmid pCDFScFbiC carry the CDF replicon, which is compatible with the pBR322 replicon of the pET22b derived expression plasmids. Transforming the pCDFScFbiC plasmid into *E. coli* cells enables the expression of the FO synthase of *S. coelicolor* (*Sc*FbiC) and consequently the biosynthesis of 8-HDF. To verify whether *Se*PhrA and *Se*PhrB could be reconstituted with 8-HDF *in vivo*, pCDFScFbiC and pETSePhrA or pETSePhrB were co-transformed into the *E. coli* BL21(DE3) cells. The culture conditions and purification procedure were the same as above, except the medium was supplemented with 50 μg ml^–1^ ampicillin and 50 μg ml^–1^ streptomycin.

### Absorption and fluorescence spectroscopy

Absorption spectra of samples were recorded on a UV-1800 spectrophotometer equipped with a TCC-240A temperature controller (Shimadzu). Semi-micro quartz cuvettes (800 μl) were used. The scanning range was from 200 to 750 nm. During recording of the spectra of the protein samples, the temperature was held at 18 ± 0.5°C.

The fluorescence spectra were recorded on a F-2700 fluorescence spectrophotometer (Hitachi). The excitation and emission slits were set to 2.5 nm. And the photomultiplier tube (PMT) voltage was fixed at 700 V. To determine an emission spectrum, the excitation wavelength was usually set to the absorption peak of the sample. To determine an excitation spectrum, the emission wavelength was usually set to the peak of the previously determined emission spectrum. If there was a discrepancy between the absorption peak and the excitation peak, another emission spectrum was measured with the excitation wavelength being set to the excitation peak of the sample. In some cases, the excitation wavelength was set to 450 nm, or the emission wavelength was set to 525 nm, to detect the existence of FAD.

The cofactors of protein samples were released by heating at neutral pH, or by acidification with HCl to pH 2.0. The protein precipitates were removed by centrifugation. The absorption and fluorescence spectra of the supernatants were monitored as above.

### Thin-layer chromatography

The supernatant samples of denatured proteins were spotted onto Type G silica gel plates (Sangon) and chromatograms were developed with three different solvent systems: (a) *n*-butanol/acetic acid/water, 5:3:2; (b) *n*-butanol/ethanol/water, 10:3:7; (c) acetonitrile/water/formic acid (88%), 40:10:5. The fluorescent spots were observed in a UV analyzer and photos were taken from the observation window.

### Photoreduction and oxidation of the proteins

The photoreduction processes of protein samples were recorded under aerobic conditions. The protein samples were in the protein buffer in the absence or presence of 10 mM DTT. The cuvette with the sample was hold in an ice-water jacket, and illuminated with a blue LED (*λ*_max_ = 440 nm). The irradiance of the LED was determined by an OHSP-350S spectral irradiance colorimeter (Hopoocolor, Hangzhou). The absorption spectra of the sample were quickly recorded at intervals.

To monitor the oxidation process, the cuvette containing the photoreduced sample was open to the air, immediately put into the UV-1800 spectrophotometer with the temperature controller set to 18 ± 0.5°C. The absorption spectra were automatically recorded at 10-min intervals.

### Determination of the 6-4 photolyase activity in vivo

To inspect the *in vivo* photorepair activity of *Se*PhrB, a previously described method (34) was used with some modification. Briefly, the *SephrB* gene was subcloned into the pTrcHisA vector (Invitrogen) to obtain pTrcSePhrB plasmid. In this plasmid the *SephrB* gene was placed under a *trc* promoter, which could be expressed in normal *E. coli* strains without T7 RNA polymerase. The *SephrA* gene was also subcloned into pTrcHisA to obtain pTrcSePhrA plasmid to give a positive reference. The pTrcSePhrA and pTrcSePhrB plasmids were respectively transformed into the *E. coli* UNC1085 strain (*recA, uvrA, phr1*). The UNC1085:pTrcSePhrA, UNC1085:pTrcSePhrB and null UNC1085 cells were grown into the stationary phase, diluted with saline (normally to 1:100), irradiated with 254 nm UVC light, and photoreactivated under a write light LED (irradiance of ∼100 W m^–2^). The induction of IPTG was omitted, because the leaky expression had already provided enough molecules of the enzymes. Before and after each treatment, aliquots of cell suspension were serially diluted, and 5-μl aliquots of dilutions were spotted on LB plates in triplicates. The colonies were counted after overnight incubation at 37°C. The survival was defined as the ratio of the colony numbers of UVC irradiated or photoreactivated samples to the non-irradiated control sample. Three independent experiments were performed for each strain under a red LED lamp.

### Determination of the 6–4 photolyase activity *in vitro*

Oligo-thymidylate (dT_16_) was dissolved in water to give a concentration of 5 μM (*A*_260 nm_ = 0.7) and irradiated with 254 nm UVC light for ∼20 000 J m^–2^. Then the substrate solution was concentrated to 0.5 mM by a Vacufuge plus concentrator (Eppendorf). This procedure produced the UV-dT_16_ substrate containing ∼three CPDs and ∼one 6–4 photoproduct per molecule. Directly irradiating oligo-thymidylate at higher concentrations was less efficient because the UV light would only be absorbed by the top layer of the solution. The activity of the protein samples was measured in a 600-μl system with 0.2 μM of protein, 10 μM UV-dT_16_, 4 mM metal ions (Mg^2+^, Mn^2+^, Ca^2+^ or K^+^) and 1 mM DTT in the protein buffer. Various LED lamps with different maximal wavelengths were used as the light sources. The slopes of absorbance decrease at 325 nm (*k*_325 nm_) were used to determine the repair activity for the 6–4 photoproducts. Because the substrate used (10 μM) was in great excess over the enzyme (0.2 μM), it could be assumed that the enzyme was fully saturated with substrate in the initial stage. This assumption should be used with caution that the light irradiance must be small enough to ensure the rate of the formation the enzyme-substrate complex (*k*_1_) not to be rate-limiting. The *k*_cat_ values were estimated by *k*_325 nm_/*ϵ*_325 nm_/[E_0_], where *ϵ*_325 nm_ was the molar extinction coefficient at 325 nm of the 6–4 photoproducts that is ∼6000 M^–1^ cm^–1^ (35); and [E_0_] was the enzyme concentration. The photolytic cross section (*ϵφ*) was calculated by using the Equation:

(1) $$\begin{equation*} \epsilon \varphi \left( {{{\rm{M}}^{ - 1}}\ {\rm{c}}{{\rm{m}}^{ - 1}}} \right) = 5.2 \times {10^8}{k_{\rm{p}}}\left( {{{\rm{m}}^{\rm{2}}}{\rm{\ }}{{\rm{J}}^{{\rm{ - 1}}}}} \right) {\lambda ^{ - 1}} \left( {{\rm{nm}}} \right) \end{equation*}$$

where *ϵ* is the molar extinction coefficient of enzyme at the illumination wavelength *λ*; *φ* is the quantum yield of photorepair; *k*_p_ is photolysis constant that equal to *k*_cat_/*L*, where *L* is the illumination dose in J m^–2^ (36). The action spectra were obtained by plotting the *ϵφ* values vs. the illumination wavelengths.

## RESULTS

### Identification of a FeS-BCP gene in the genome of *S. elongatus*

Photolyases and their homologs cryptochromes constitute a large protein family called cryptochrome/photolyase family (CPF). Our phylogenetic work shows that CPF proteins are divided into eight main groups: class I CPD photolyases, class III CPD photolyases (including plant cryptochromes), DASHs, DASH-likes, eukaryotic 6–4 photolyases (including animal cryptochromes), class II CPD photolyases, prokaryotic 6–4 photolyases (including FeS-BCPs), and SPLs (Figure 1A) (25). From the whole genome shotgun sequences of *S. elongatus* PCC 7942 (28), three CPF genes were identified: *SephrA* encodes a class I CPD photolyase; and *SephrB* and *SephrC* encode a FeS-BCP and a SPL, respectively (Figure 1A). Protein sequence alignment analysis showed that *Se*PhrB has identities of 43.14% (220/510) and 41.96% (201/479) to *A. fabrum* FeS-BCP (*Af*PhrB) and *Rs*CryB, respectively (Figure 1B).

To clarify the relationships of *Se*PhrB and other prokaryotic 6–4 photolyase members, and to obtain more phylogenetic information about prokaryotic 6–4 photolyases, we performed a comprehensive analysis with 110 prokaryotic 6–4 photolyase sequences from various species, including *Af*PhrB (17,18), *Rs*CryB (19,20), *V. cholerae* FeS-BCP (*Vc*FeS-BCP) (21), *Sphingomonas* sp. FeS-BCP (*Sph*PhrB) (22), *P. marinus* PromaPL (24), and *Se*PhrB. Prokaryotic 6–4 photolyases were divided into seven subgroups (Figure 1C and Supplementary Figure S1). Three subgroups were named ‘A’, ‘B’, and ‘G’, because the major members of which are from the classes *Alphaproteobacteria* (18/26), *Betaproteobacteria* (6/12), and *Gammaproteobacteria* (13/21), respectively. These subgroups also form a large cluster ‘ABG’. *Bacillus chagannorensis* FeS-BCP lies outside of the cluster ABG but does not belong to any subgroups. A small subgroup was named ‘Chl’, which is constituted with three members from the phylum *Chloroflexi*, one from the phylum *Chlorobi*, and one from the phylum *Acidobacteria*. The subgroup ‘HC’ is mainly constituted with the members from the class *Halobacteria* (6/12) and the phylum *Cyanobacteria* (4/12). Members of the subgroup ‘Ac’ are from the phyla *Actinobacteria* (10/13) and *Acidobacteria* (3/13). The last subgroup was named ‘M’, because the members of which (20 in total, including *P. marinus* PromaPL) are from multiple lineages. Intriguingly, it was observed that all prokaryotic 6–4 photolyases except for the members of the subgroup M contain the four conserved cysteines that may coordinate an iron-sulfur cluster (18,19), which can be catalogued as the FeS-BCP subtype.

It was found that *Af*PhrB, *Rs*CryB and *Sph*PhrB have high homology, all of which belong to the subgroup A. *Vc*FeS-BCP is a member of the subgroup G. All these four FeS-BCPs are from the cluster ABG. Therefore, it is not surprising that they share many features. However, *Se*PhrB is a member of the subgroup HC, which is distantly related to the previously described FeS-BCPs. It was expected that *Se*PhrB might have some unique properties.

### *E. coli* expressed *Se*PhrB has no DMRL antenna cofactor

**Figure 2.**
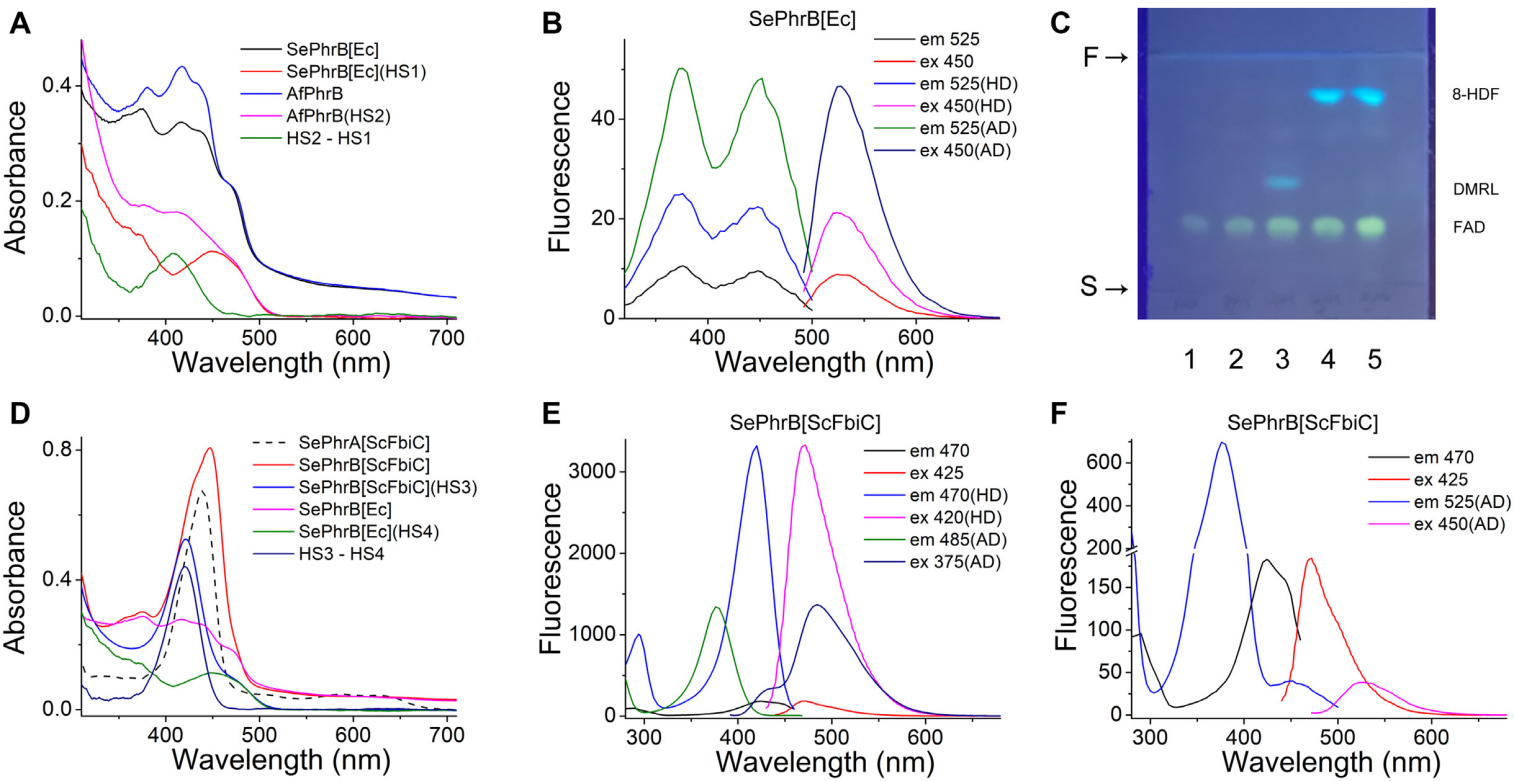
The spectroscopy and thin-layer chromatography analyses of *Se*PhrB and other proteins. (**A**) The absorption spectra of *E. coli* expressed *Se*PhrB (*Se*PhrB[*Ec*]) and *Af*PhrB. HS1 and HS2 are the spectra of the supernatants of heat denatured *Se*PhrB[*Ec*] and *Af*PhrB, respectively. And HS2 – HS1 is the difference spectrum of HS2 and HS1. (**B**) The emission and excitation fluorescence spectra of native *Se*PhrB[*Ec*], and the supernatants of heat denatured (HD) or acid denatured (AD) *Se*PhrB[*Ec*]. (**C**) The thin-layer chromatography of authentic FAD (lane 1), and the supernatants of heat denatured *Se*PhrB[*Ec*] (lane 2), *Af*PhrB (lane 3), *Se*PhrA co-expressed with *Sc*FbiC (*Se*PhrA[*Sc*FbiC], lane 4), and *Se*PhrB co-expressed with *Sc*FbiC (*Se*PhrB[*Sc*FbiC], lane 5). The solvent system was *n*-butanol/acetic acid/water, 5:3:2. The start and front positions (S and F) are indicated by arrows. (**D**) The absorption spectra of *Se*PhrA[*Sc*FbiC], *Se*PhrB[*Sc*FbiC], and *Se*PhrB[*Ec*]. HS3 and HS4 are the spectra of the supernatants of heat denatured *Se*PhrB[*Sc*FbiC] and *Se*PhrB[*Ec*], respectively. And HS3 – HS4 is the difference spectrum of HS3 and HS4. (**E**) The emission and excitation fluorescence spectra of native *Se*PhrB[*Sc*FbiC], and the supernatants of heat denatured (HD) or acid denatured (AD) *Se*PhrB[*Sc*FbiC]. (**F**) The magnified fluorescence spectra of native *Se*PhrB[*Sc*FbiC], and the spectra of the supernatants of acid denatured (AD) *Se*PhrB[*Sc*FbiC] with the excitation wavelength set at 450 nm, and the emission wavelength set at 525 nm to detect the existence of FAD. The protein concentrations used in the absorption spectroscopy analyses were ∼10 μM, and those used in fluorescence spectroscopy analyses were ∼2 μM.

To express *Se*PhrB in *E. coli*, the *SephrB* gene was cloned into the pET22b vector; and the resulting plasmid pETSePhrB was transformed into *E. coli* BL21(DE3) cells. *Af*PhrB was also expressed in *E. coli* in a similar way to give a comparison. The absorption spectrum of purified *Se*PhrB expressed in *E. coli* (hereafter referred to as *Se*PhrB[*Ec*]) exhibited peaks at 375 and 417 nm, and shoulders at 440 and 470 nm (Figure 2A, black line), which was similar to that of *Af*PhrB (Figure 2A, blue line) (17). The broad absorption extending beyond 700 nm suggested that *Se*PhrB[*Ec*] possesses an iron-sulfur cluster as *Af*PhrB (17,18). Indeed, chemical analyses revealed that one mole of *Se*PhrB[*Ec*] contained ∼3.5 mol of iron and ∼4.2 mol acid-labile sulfide (Supplementary Figure S2A and B).

However, the absorbance of *Se*PhrB[*Ec*] in the 350–450 nm range was significantly lower than that of *Af*PhrB. The supernatant of heat denatured *Af*PhrB showed multiple peaks in the 300–500 nm range (Figure 2A, magenta line). In contrast, *Se*PhrB[*Ec*] exhibited an obvious absorption peak at 450 nm after being heat denatured and centrifugated (Figure 2A, red line). Moreover, the difference spectrum of the supernatant of *Af*PhrB minus that of *Se*PhrB[*Ec*] gave a peak at 410 nm, which is a characteristic of DMRL (Figure 2A, olive line) (37). These results suggested that *Se*PhrB[*Ec*] contains a flavin cofactor without a second cofactor, or with a second cofactor which had been decomposed after heat denaturation in neutral conditions, such as methenyltetrahydrofolate (MTHF) (38).

The fluorescence excitation and emission spectra of *Se*PhrB[*Ec*] were determined, which exhibited two excitation peaks at 370 and 450 nm, and an emission peak at 525 nm (Figure 2B, black and red lines). When the sample was heat denatured and centrifugated, the fluorescence increased about 2 folds (Figure 2B, blue and magenta lines). When *Se*PhrB[*Ec*] was denatured by acidification to pH 2.0, a ∼5-fold increase of the fluorescence intensity was observed (Figure 2B, olive and navy lines). These results were characteristic for protein-bound FAD and ruled out the possibility that the protein associated with a MTHF cofactor.

In comparison, the fluorescence excitation and emission spectra of *Af*PhrB show an excitation peak at 405 nm, and an emission peak at 475 nm (Supplementary Figure S3A, black and red lines). After being heat denatured and centrifugated, the fluorescence of *Af*PhrB increased more than 10 folds and the emission peak was shifted to 485 nm, which was mainly due to the released DMRL cofactor (Supplementary Figure S3A, blue and magenta lines). Nevertheless, when *Af*PhrB was acid-denatured at pH 2.0, the fluorescence of released DMRL was partially quenched, and that of FAD was intensified compared to those released at neutral conditions. The excitation and emission spectra of acid-denatured *Af*PhrB could be roughly divided into DMRL and FAD components (Supplementary Figure S3A, olive and navy lines; and Supplementary Figure S3B).

The supernatant samples of denatured *Se*PhrB[*Ec*] and *Af*PhrB were analyzed by thin-layer chromatography. The supernatant of *Se*PhrB[*Ec*] gave only one yellow fluorescent band with the same *R*_f_ value of authentic FAD (Figure 2C, lanes 2 and 1; and Supplementary Figure S4). Meanwhile, the supernatant of *Af*phrB produced a yellow fluorescent band of FAD, and a blue-green fluorescent band of DMRL (Figure 2C, lane 3; and Supplementary Figure S4). Therefore, we concluded that *E. coli* expressed *Se*PhrB do not possess a DMRL antenna cofactor as the other reported FeS-BCPs.

### SePhrB associates with 8-HDF when co-expressed with the *S. coelicolor* FO synthase

The deazariboflavin cofactor 8-HDF, or named FO, is employed by a number of class I/III CPD photolyases (including *Se*PhrA) (7,39), class II CPD photolyases (27), and eukaryotic 6–4 photolyases/bifunctional cryptochromes (26,40–43), to sever as an antenna cofactor that harvests and transfers more light energy to the catalytic FAD cofactor to enhance the photorepair activity of the enzymes. The synthesis of 8-HDF requires a FO synthase that is composed of two subunits CofG and CofH, or a fusion protein FbiC with two domains that are homologous to CofG and CofH (44). Genome sequence analysis showed that *S. elongatus* PCC 7942 contains a *cofG* gene (H6G84_11875) and a *cofH* gene (H6G84_12985). However, *E. coli* lacks FO synthase, therefore do not synthesize 8-HDF.

To enable the synthesis of 8-HDF in *E. coli*, a method was adapted from a previous study (27). The *S. coelicolor fbiC* gene was cloned into the pCDFDuet-1 vector. The resulting plasmid pCDFScFbiC and the plasmid pETSePhrB were transformed together into *E. coli* BL21(DE3) cells to co-express the *S. coelicolor* FO synthase (*Sc*FbiC) and *Se*PhrB. To check whether 8-HDF was properly synthesized and capable of binding to target proteins, *Se*PhrA was co-expressed with *Sc*FbiC in a similar way. After purification, *Se*PhrA co-expressed with *Sc*FbiC gave a prominent absorption peak at 439 nm (Figure 2D, black dash line, referred to as *Se*PhrA[*Sc*FbiC]) that corresponded closely to the previously reported spectra (7,14,27), implying that 8-HDF had been successfully incorporated into the protein. Meanwhile, compared with *Se*PhrB[*Ec*] (Figure 2A, black line; and Figure 2D, magenta line), purified *Se*PhrB co-expressed with *Sc*FbiC (referred to as *Se*PhrB[*Sc*FbiC]) exhibited an additional absorption peak at 447 nm (Figure 2D, red line). The supernatant of heat denatured *Se*PhrB[*Sc*FbiC] showed an absorption peak at 420 nm and a shoulder at 475 nm (Figure 2D, blue line). After subtraction of the absorption spectrum of heat denatured *Se*PhrB[*Ec*] (Figure 2D, olive line) from that of heat denatured *Se*PhrB[*Sc*FbiC], a single absorption peak at 420 nm was obtained, which was identical to that of 8-HDF (Figure 2D, navy line).

The fluorescence spectra of *Se*PhrB[*Sc*FbiC] had an emission peak at 470 nm with an excitation maximum at 425 nm and a shoulder at 445 nm (Figure 2E and magnified in Figure 2F, red and black lines). When *Se*PhrB[*Sc*FbiC] was heat denatured, the fluorescence intensity of the supernatant dramatically increased ∼18 folds, and the excitation peak was shifted to 420 nm (Figure 2E, blue and magenta lines). The fluorescence intensity of *Se*PhrB[*Sc*FbiC] denatured at pH 2.0 was only ∼40% compared to that of heat denatured one; and the excitation and emission peaks were shifted to 375 nm and 485 nm, respectively (Figure 2E, olive and navy lines). The fluorescence properties of the heat- and acid-denatured samples corresponded well to those of 8-HDF. To prove the existence of FAD, the supernatant of *Se*PhrB[*Sc*FbiC] denatured at pH 2.0 was excited at 450 nm, in which conditions 8-HDF was not excited, a relatively weak emission peak at 525 nm was obtained (Figure 2F, magenta line); when the emission spectrum was measured at 525 nm, a small excitation peak at 450 nm (corresponding to FAD) and a large peak at 375 nm (corresponding to 8-HDF) were observed (Figure 2F, blue line).

Thin-layer chromatography analyses showed that the supernatant of *Se*PhrA[*Sc*FbiC] and that of *Se*PhrB[*Sc*FbiC] gave identical results: each of them had a yellow FAD band, and an additional bright blue band that was attributed to 8-HDF (Figure 2C, lanes 4 and 5; Supplementary Figure S4). These results demonstrated that *Se*PhrB associates with FAD and 8-HDF when co-expressed with the *Sc*FbiC. Hereafter *Se*PhrB[*Sc*FbiC] was referred to as *Se*PhrB[8-HDF]. According to the molar extinction coefficients of free FAD (*ϵ*_450 nm_ = 11 300 M^–1^ cm^–1^) and 8-HDF (*ϵ*_400 nm_ = 25 700 M^–1^ cm^–1^) (45), it was calculated that *Se*PhrB[8-HDF] contained equimolar amounts of the two cofactors from the absorption spectrum of denatured protein (Figure 2D, blue line).

### Photoreduction and oxidation of *Se*PhrB[*Ec*]

Photolyases require fully reduced FAD to perform their DNA repair functions. The FAD cofactor in photolyases in the fully oxidized or radical state could be photoreduced to the catalytic active, full reduced state in the presence of external electron donors (46,47). Inspection of the absorption and fluorescence spectra of purified *Se*PhrB[*Ec*] (Figure 2A, black line; and Figure 2B, black and red lines) suggested that the protein contained fully oxidized FAD.

As previously reported (17), it was found that *Af*PhrB could be photoreduced under blue light (*λ*_max_ = 440 nm, irradiance of ∼190 W m^–2^) in the presence of 10 mM DTT (Supplementary Figure S5A and S5B). Because the formation of neutral radical state FAD was negligible during the photoreduction process, the photoreduction of *Af*PhrB could be roughly regarded as a first order reaction. By mono-exponential fitting, the pseudo photoreduction rate constant (*k*_pr_) of *Af*PhrB in the presence of DTT was obtained to be 1.4 ± 0.3 × 10^–3^ s^–1^ (Supplementary Figure S5D). In the absence of DTT, photoreduction of *Af*PhrB was very slow (*k*_pr_ of 6.2 ± 1.3 × 10^–5^ s^–1^), and only a small fraction of the protein was photoreduced after extensive illumination under blue light (Supplementary Figure S5C and S5D).

**Figure 3.**
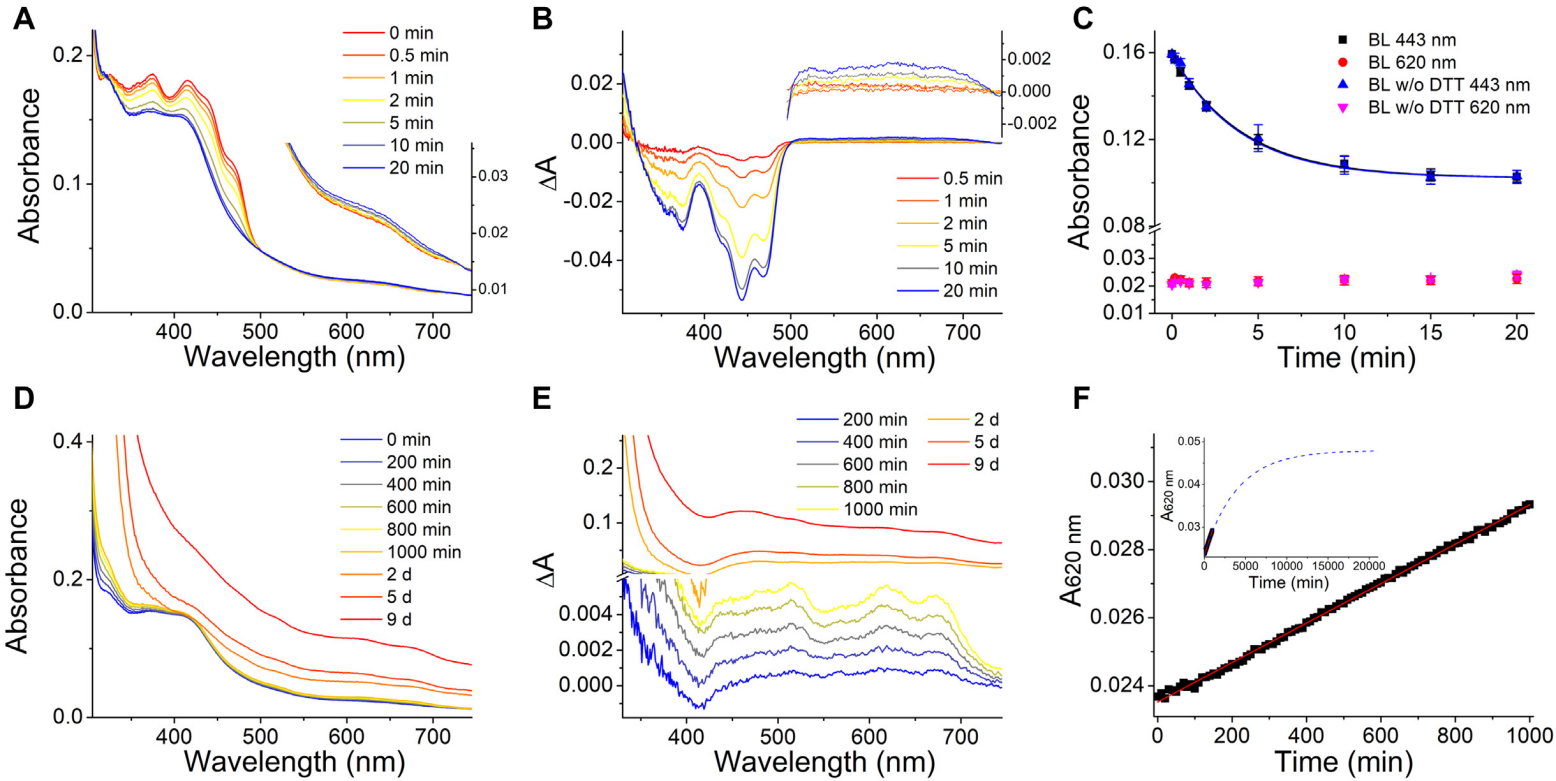
Photoreduction and oxidation of *Se*PhrB[*Ec*]. (**A**) The *Se*PhrB[*Ec*] sample (5.3 μM) was illuminated under blue light (*λ*_max_ = 440 nm, irradiance of ∼190 W m^–2^) for 20 min without DTT. The absorption spectra were recorded at indicated intervals. The inset shows the magnified spectra in the 530–750 nm range. The results of the experiments with 10 mM DTT are almost identical, which are not shown for clarity. (**B**) The difference spectra of illuminated and non-illuminated *Se*PhrB[*Ec*] calculated from the data shown in (A). The inset shows the magnified spectra in the 500–750 nm range. (**C**) The photoreduction kinetics of *Se*PhrB[*Ec*] under the blue light with 10 mM and without DTT depicted using the absorbance changes at the peak of fully oxidized FAD (443 nm) and at that of neutral radical FAD (620 nm) in *Se*PhrB[*Ec*]. Data points represent means ± SD (*n* = 3). The absorbance changes at 620 nm were relatively small that could be negligible. And the absorbance changes at 443 nm were fitted with a mono-exponential function to obtain pseudo photoreduction rate constants (*k*_pr_) to be 4.1 ± 0.4 × 10^–3^ s^–1^ and 4.2 ± 0.9 × 10^–3^ s^–1^ for the *Se*PhrB[*Ec*] samples with and without DTT, respectively. The *R*^2^ values of the fittings were 0.999 and 0.997, respectively. (**D**) The photoreduced *Se*PhrB[*Ec*] sample was oxidized in the dark under aerobic conditions at 18 ± 0.5°C. The absorption spectra were recorded at indicated intervals. (**E**) The difference spectra of oxidizing and just photoreduced *Se*PhrB[*Ec*] calculated from the data shown in (**D**). (**F**) The oxidation kinetics of *Se*PhrB[*Ec*] depicted using the absorbance change at the peak of neutral radical FAD (620 nm). The oxidation rate constant from the fully reduced state to the neutral radical state (*k*_ox1_) value was calculated to be 3.5 ± 0.8 × 10^–6^ s^–1^ by linear fitting. The inset shows the predicted oxidation process assuming the oxidation of *Se*PhrB[*Ec*] from the fully reduced state to the neutral radical state was a first order reaction.

To our surprise, we found that *Se*PhrB[*Ec*] could be photoreduced under blue light in the absence of external electron donors (Figure 3A). The difference spectra of illuminated and non-illuminated *Se*PhrB[*Ec*] showed two negative bands at 375 nm and 443 nm, and shoulders at 355, 419 and 468 nm, which was assigned to photoreduction of oxidized FAD (Figure 3B). Nevertheless, a weak absorption increase at 500–750 nm was observed, suggesting the formation of a small amount of neutral radical FAD (Figure 3B). The spectrum of *Se*PhrB[*Ec*] after 20 min blue light illumination exhibited a lower peak at 375 nm and a shoulder at 410 nm, which indicated that most of the protein was photoreduced to the fully reduced state (Figure 3A). It was calculated that the *k*_pr_ values of *Se*PhrB[*Ec*] under the blue light in the absence and presence of 10 mM DTT were 4.2 ± 0.9 × 10^–3^ s^–1^ and 4.1 ± 0.4 × 10^–3^ s^–1^, respectively (Figure 3C). These results demonstrated that DTT had little effect on the photoreduction of *Se*PhrB[*Ec*].

It was observed that photoreduced *Af*PhrB was almost completely oxidized within ∼400 min when kept in the dark under aerobic conditions, with the oxidation rate constant from the fully reduced state to the neutral radical state (*k*_ox1_) of 2.6 ± 0.3 × 10^–4^ s^–1^, and that from the neutral radical state to the fully oxidized state (*k*_ox2_) of 1.5 ± 0.6 × 10^–4^ s^–1^ (Supplementary Figure S5E and S5F). In contrast, the oxidation of photoreduced *Se*PhrB[*Ec*] was very slow. After incubation of photoreduced *Se*PhrB[*Ec*] in the dark under aerobic conditions for 1000 min, there was only a slight absorption increase over broad spectral range (Figure 3D). The difference spectra showed no absorption peak around 450 nm, implying that oxidized FAD was not formed. A negative peak at ∼410 nm was observed, which might be due to the oxidation of fully reduced FAD and/or the decomposition of the iron-sulfur cluster. The formation of neutral radical FAD was demonstrated by the absorption maxima at 620 and 674 nm (Figure 3E). These peaks were significantly red-shifted compared with those of *Af*PhrB (at 584 nm and 630 nm, Supplementary Figure S5B), indicating that the FAD binding environments were different in these two proteins. The *k*_ox1_ value was calculated to be 3.5 ± 0.8 × 10^–6^ s^–1^ by linear fitting (Figure 3F), which was approximately two-order lower than that of *Af*PhrB. Assuming the oxidation of *Se*PhrB[*Ec*] from the fully reduced state to the neutral radical state was a first order reaction, it was estimated that it would take 15 000–20 000 min to reach a plateau (Figure 3F, inset). The *k*_ox2_ value of *Se*PhrB[*Ec*] could not be obtained, because even after 9 days incubation in the dark, there was no significant amount of fully oxidized state FAD formed, although slight protein aggregation already occurred (Figure 3D and E).

### Photoreduction and oxidation of *Se*PhrB[8-HDF]

**Figure 4.**
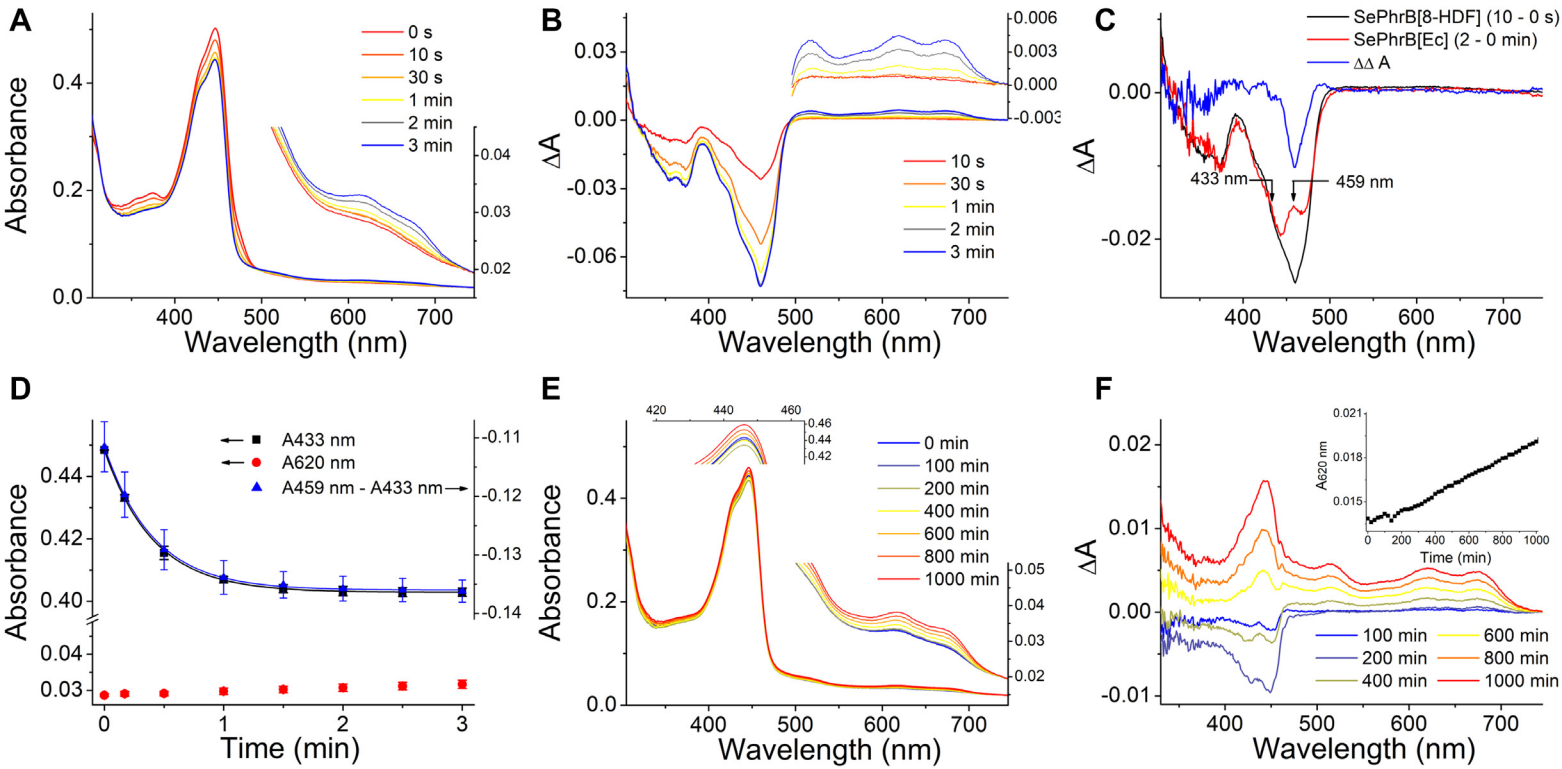
Photoreduction and oxidation of *Se*PhrB[8-HDF]. (**A**) The *Se*PhrB[8-HDF] sample (6.2 μM) was illuminated under blue light (*λ*_max_ = 440 nm, irradiance of ∼190 W m^–2^) for 3 min without DTT. The absorption spectra were recorded at indicated intervals. The inset shows the magnified spectra in the 510–750 nm range. (**B**) The difference spectra of illuminated and non-illuminated *Se*PhrB[8-HDF] calculated from the data shown in (A). The inset shows the magnified spectra in the 500–750 nm range. (**C**) Double difference spectrum made by subtracting the light-induced difference spectrum of *Se*PhrB[*Ec*] (2–0 min) from that of *Se*PhrB[8-HDF] (10–0 s) after scaling the 375 nm bands. The resulting spectrum exhibits a negative peak at 459 nm. (**D**) The photoreduction kinetics of *Se*PhrB[8-HDF] under the blue light depicted using the absorbance change at 433 nm (reflecting the photoreduction of fully oxidized FAD) and that at 620 nm (reflecting the formation of neutral radical FAD), and the difference between the absorbance change at 459 nm and that at 433 nm (reflecting the development of the negative peak at 459 nm). Data points represent means ± SD (*n* = 3). The absorbance change at 620 nm was relatively small that could be negligible. The absorbance change at 433 nm was fitted with a mono-exponential function to obtain *k*_pr_ of FAD photoreduction to be 4.1 ± 0.6 × 10^–2^ s^–1^ (*R*^2^ = 1.000). And the rate constant of the development of the negative peak at 459 nm was obtained to be 4.1 ± 1.3 × 10^–2^ s^–1^ by mono-exponential fitting of the difference between the absorbance change at 459 nm and that at 433 nm (*R*^2^ = 0.999). (**E**) The photoreduced *Se*PhrB[8-HDF] sample was oxidized in the dark under aerobic conditions at 18 ± 0.5°C. The absorption spectra were recorded at indicated intervals. The insets show the magnified spectra in the 420–465 and 500–750 nm ranges. (**F**) The difference spectra of oxidizing and just photoreduced *Se*PhrB[8-HDF] calculated from the data shown in (**E**). The inset shows the oxidation kinetics of *Se*PhrB[8-HDF] depicted using the absorbance change at the peak of neutral radical FAD (620 nm). The *k*_ox1_ value was calculated to be 3.8 ± 1.0 × 10^–6^ s^–1^ by linear fitting.

For *Se*PhrB[8-HDF], the high absorption and fluorescence of 8-HDF hampered determination of the redox state of FAD. But the similar absorption peak of purified *Se*PhrB[8-HDF] at 375 nm (Figure 2D, red line) compared with that of *Se*PhrB[*Ec*] (Figure 2D, magenta line) implied that their FAD redox states should be the same. The photoreduction of *Se*PhrB[8-HDF] was much faster, which was photoreduced to a metastable state within 3 min under the same illumination conditions as above (Figure 4A). The illuminated minus non-illuminated difference spectra of *Se*PhrB[8-HDF] were similar to those of *Se*PhrB[*Ec*] but with several differences (Figure 4B). Positive bands at 620 nm and 674 nm were observed, which were indicative of the formation of neutral radical FAD. These bands of *Se*PhrB[8-HDF] were still weak after illuminated for 3 min, but they were more prominent than those of *Se*PhrB[*Ec*] after illuminated for 20 min. A negative band at 375 nm was also observed, indicating the photoreduction of oxidized state FAD; but another negative band was shifted to 460 nm. To elucidate the reason for the band-shift, double difference spectrum was made by subtracting the light-induced difference spectrum of *Se*PhrB[*Ec*] (2–0 min) from that of *Se*PhrB[8-HDF] (10–0 s) after scaling the 375 nm bands. The resulting spectrum exhibits a negative peak at 459 nm (Figure 4C). This was reminiscent of a recently report that during the red-light photoreduction of *Chlamydomonas reinhardtii* aCRY (a bifunctional cryptochrome) with 8-HDF from the neutral radical state to the fully reduced state, a positive peak at 458 nm was observed in the illuminated minus non-illuminated difference spectra, which was interpreted as a result of 8-HDF deprotonation (42). It was reported that protein binding environments and the protonation states of 8-HDF affect its absorption maximum and intensity (43). Therefore, the negative band at 459 nm might reflect subtle changes in the protein binding environments and the protonation states of 8-HDF upon illumination.

Considering the light-induced difference spectrum of *Se*PhrB[*Ec*] could represent photoreduction of FAD from the fully oxidized state to the fully reduced state, we could depict the photoreduction process of FAD in *Se*PhrB[8-HDF] by using the absorbance change at any wavelength that superimposed to the difference spectrum of *Se*PhrB[*Ec*]. It was found that the difference spectra of *Se*PhrB[*Ec*] and *Se*PhrB[8-HDF] were superimposed at 433 nm. Nevertheless, the absorbance at 433 nm was identical to that at 459 nm in the difference spectrum of *Se*PhrB[*Ec*] (Figure 4C). Therefore, we analyzed the photoreduction process of FAD in *Se*PhrB[8-HDF] by the absorbance change at 433 nm; and investigated the development of the negative peak at 459 nm by the difference between the absorbance change at 459 nm and that at 433 nm. The *k*_pr_ of FAD photoreduction and the rate constant of the development of the negative peak at 459 nm were obtained by mono-exponential fitting, which were 4.1 ± 0.6 × 10^–2^ s^–1^ and 4.1 ± 1.3 × 10^–2^ s^–1^, respectively (Figure 4D). This result suggested that the two processes were closely linked. The photoreduction of FAD in *Se*PhrB[8-HDF] was ∼10-fold faster than that in *Se*PhrB[*Ec*], demonstrating that the presence of 8-HDF greatly facilitated the photoreduction of FAD. Because the extinction coefficient of 8-HDF is less than 10-fold of that of the oxidized state FAD, this result implies that the energy transferred from 8-HDF is more efficiently utilized to induce photoreduction than that directly absorbed by FAD. The amount of neutral radical FAD formed during short photoreduction process was relatively small (Figure 4D). Nevertheless, it was observed that prolonged illumination of *Se*PhrB[8-HDF] caused formation of a larger amount of neutral radical FAD, which might be due to photo-induced oxidation of fully reduced FAD. After that, neutral radical FAD was gradually photoreduced again together with 8-HDF, leaving a low absorption shoulder at 447 nm (Supplementary Figure S6). However, we found that this extreme treatment led to completely loss of activity of the protein, which seemed to have little physiological significance, therefore was not investigated further in this study.

The oxidation of shortly photoreduced *Se*PhrB[8-HDF] was a bit complicated. It was observed that the absorbance at 400–460 nm decreased during first 200 min, then gradually increased up to 1000 min (Figure 4E and F). This change could not be attributed to the producing of fully oxidized FAD, because the characteristic peak at 375 nm and the shoulder at 470 nm of oxidized FAD were not observed. We speculated that it might be due to some hypochromic and hyperchromic effects on 8-HDF during dark incubation. The absorption peaks at 620 and 674 nm raised slowly, indicating that the fully reduced FAD cofactor was oxidized to the neutral radical state (Figure 4E and F). Linear fitting showed that the *k*_ox1_ value of shortly photoreduced *Se*PhrB[8-HDF] was 3.8 ± 1.0 × 10^–6^ s^–1^ (Figure 4F, inset), which was almost identical to that of photoreduced *Se*PhrB[*Ec*].

### SePhrB has 6-4 photolyase activity both *in vivo* and *in vitro*

**Figure 5.**
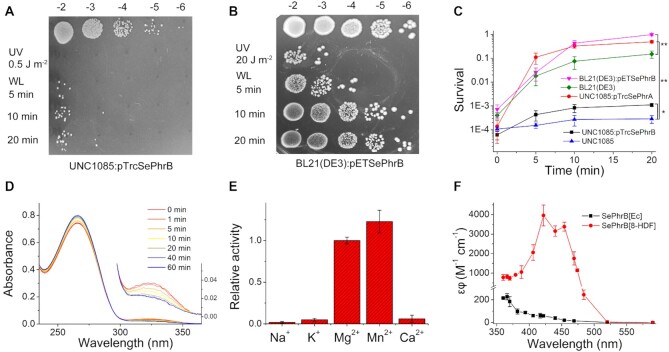
Determination of the 6–4 photolyase activity of *Se*PhrB *in vivo* and *in vitro*. (**A**) The expression of *Se*PhrB complemented the photorepair defection of UNC1085 (*recA, uvrA, phr1*). The pTrcSePhrB plasmid harboring the *SephrB* gene under the *trc* promoter was transformed into the UNC1085 strain. The resulting UNC1085:pTrcSePhrB strain was given a UV dose of ∼0.5 J m^–2^, and photoreactivated under white light (irradiance of ∼100 W m^–2^). Tens of times of survival increase was observed after illumination for 20 min. (**B**) The expression of *Se*PhrB improved the photorepair capacity of BL21(DE3). The pETSePhrB plasmid harboring the *SephrB* gene under the T7 promoter was transformed into the BL21(DE3) strain. The resulting BL21(DE3):pETSePhrB strain was given a UV dose of ∼20 J m^–2^, and photoreactivated under white light (irradiance of ∼100 W m^–2^). Nearly 100% photoreactivation was observed after illumination for 20 min. (**C**) Comparison of photoreactivation rates of five strains. The UNC1085, UNC1085:pTrcSePhrA, and UNC1085:pTrcSePhrB strains were given a UV dose of ∼0.5 J m^–2^. And The BL21(DE3) and BL21(DE3):pETSePhrB strains were given a UV dose of ∼20 J m^–2^. The survival rates of these strains were determined after white light illumination for indicated periods of time. Data points represent means ± SEM (n = 3). *P < 0.05, **P < 0.01. (**D**) A representative photorepair reaction of *Se*PhrB for 6–4 photoproducts in UV-irradiated oligo-thymidylate (UV-dT_16_). The activity was measured in a 600-μl system with 0.2 μM of *Se*PhrB[*Ec*], 10 μM UV-dT_16_, 4 mM MgCl_2_, and 1 mM DTT in the protein buffer. A UVA LED lamps (*λ*_max_ = 370 nm, irradiance of ∼77 W m^–2^) were used as the light sources. The 6–4 photolyase activity is demonstrated by the absorbance decrease at 325 nm (also shown in the inset) and the mutual absorbance increase at 265 nm. (**E**) The 6–4 photolyase activity of *Se*PhrB with different metal ions. Data represent means ± SD (*n* = 3). (**F**) The action spectra of *Se*PhrB[*Ec*] and *Se*PhrB[8-HDF] for photorepair 6–4 photoproducts *in vitro*. Data points represent means ± SD (*n* = 3).

To verify whether *Se*PhrB has photolyase activity *in vivo*, two plasmids named pTrcSePhrA and pTrcSePhrB were constructed, in which the *SephrA* and *SephrB* genes were inserted into the pTrcHisA vector under the control of the *trc* promoter. The two plasmids were respectively transformed into the *E. coli* UNC1085 strain (*recA, uvrA, phr1*). The null UNC1085 strain was set as the negative control. After giving a UV dose of ∼0.5 J m^–2^, the survival rates of all strain cells decreased to ∼10^–4^. Then the cells were photoreactivated under white light (irradiance of ∼100 W m^–2^). The UNC1085 strain only exhibited residual photoreactivation after white light illumination for 20 min. In contrast, the survival rates of the UNC1085:pTrcSePhrA and UNC1085:pTrcSePhrB strains increased thousands and tens of times after illumination, respectively (Figure 5A and C). The photoreactivation of the BL21(DE3):pETSePhrB strain was also investigated. The BL21(DE3) strain contains an intact CPD photolyase gene. After UV irradiation of 20 J m^–2^ UV and the white light illumination for 20 min, about 10% of the null BL21(DE3) could be photoreactivated; while nearly 100% photoreactivation of the BL21(DE3):pETSePhrB cells was observed (Figure 5B and C). These results revealed that *Se*PhrB could photorepair a smaller fraction of UV-induced DNA lesions other than CPDs, which was expected to be 6–4 photoproducts.

The 6–4 photolyase activity of *Se*PhrB was also investigated *in vitro*. UV-irradiated oligo-thymidylate (UV-dT_16_) was used as the substrate that contained both CPDs (∼3 per molecule) and 6–4 photoproducts (∼1 per molecule). It was observed that *Se*PhrB[*Ec*] was capable of photorepairing 6–4 photoproducts in DNA efficiently under UVA light (*λ*_max_ = 370 nm, irradiance of ∼77 W m^–2^) in the presence of Mg^2+^, which was demonstrated by the gradual decrease of the absorbance at 325 nm and the mutual increase of the absorbance at 265 nm (Figure 5D). As previously reported FeS-BCPs (48), the 6–4 photolyase activity of *Se*PhrB was dependent on divalent metal ions, such as Mg^2+^ and Mn^2+^; but Ca^2+^ and the monovalent metal ions Na^+^ and K^+^ had little effect on stimulating the activity (Figure 5E). The photorepair velocity of *Se*PhrB[*Sc*FbiC] was significantly faster than that of *Se*PhrB[*Ec*] at all illumination wavelengths. The photolytic cross section (*ϵφ*) values of *Se*PhrB[*Ec*] and *Se*PhrB[8-HDF] were plotted vs. the illumination wavelengths to obtain the action spectra (Figure 5F). The action spectrum of *Se*PhrB[*Ec*] resembles the absorption spectrum of the fully reduced FAD. The *ϵφ* value at 366 nm was 227 M^–1^ cm^–1^. Assuming the *ϵ*_366 nm_ of the fully reduced FAD in *Se*PhrB[*Ec*] was ∼6,000 M^–1^ cm^–1^, and the absorption of iron-sulfur cluster had no contribution to the reaction, the photorepair quantum yield of *Se*PhrB[*Ec*] at 366 nm was estimated to be ∼0.04, which corresponded well with other 6–4 photolyases (49,50). The *ϵφ* values of *Se*PhrB[8-HDF] were 3.6-fold (at 370 nm) to 169-fold (at 454 nm) higher than those of *Se*PhrB[*Ec*], indicating that the presence of 8-HDF greatly elevated the repair activity of the enzyme. The maximal *ϵφ* of *Se*PhrB[8-HDF] was observed at 420 nm, which was ∼4000 M^–1^ cm^–1^. Assuming the *ϵ*_420 nm_ of reduced *Se*PhrB[8-HDF] excluding the iron-sulfur cluster (only taking account of 8-HDF + fully reduced FAD) was ∼40 000 M^–1^ cm^–1^, the photorepair quantum yield would be ∼0.1, implying that the presence of 8-HDF also increased the quantum yields of the reaction. It was interesting that the maximum of *ϵφ* was not at the absorption peak of *Se*PhrB[8-HDF] (447 nm). Instead, it seemed that there was a small valley at ∼440 nm in the action spectrum of *Se*PhrB[8-HDF], which might be due to photo-induced change of 8-HDF that inhibited the energy transferring from 8-HDF to FAD.

## DISCUSSION

Cyanobacteria are one of the oldest living organisms on the earth. And they are obligate photoautotrophs, which cannot live without sunlight in the nature. Therefore, cyanobacteria are also inevitably suffering from the detrimental effects of the UV component of sunlight on cellular DNA. Many cyanobacteria have efficient photoreactivation ability, by which UV-induced DNA lesions (CPDs and 6–4 photoproducts) are directly recovered using external light energy. For example, *S. elongatus* could be recovered up to 100% survival by photoreactivation even after extensive UV irradiation (11). However, for a long time only one CPD photolyase (*Se*PhrA) was discovered in *S. elongatus*, which had been extensively studied as a model of CPD photolyases (7,12–16). In this work, a prokaryotic 6–4 photolyase (*Se*PhrB) of *S. elongatus* was identified and characterized, which was demonstrated to be able to efficiently photorepair 6–4 photoproducts both *in vivo* and *in vitro*. This finding could explain the observation of the full photoreactivation of *S. elongatus* after UV inactivation.

Phylogenetic analyses revealed that *Se*PhrB is a member of FeS-BCPs, a subtype of prokaryotic 6–4 photolyases. A characteristic of FeS-BCPs is that they have an iron-sulfur cluster (18,19,21,22). The absorption spectroscopy and chemical evidence suggested that *Se*PhrB also contained an iron-sulfur cluster as the other reported FeS-BCPs. It was found that the iron-sulfur cluster is required for structural stability, substrate binding, and repair activity of *Af*PhrB (18,51,52). The iron-sulfur cluster in *Se*PhrB might have the similar roles. The iron-sulfur cluster had broad absorption in the 300–700 nm range and a maximum at 400–420 nm. It was an interesting question whether the light absorbed by the iron-sulfur cluster could facilitate the photorepair reaction of FeS-BCPs. However, the shape of the action spectrum of *Se*PhrB[*Ec*] resembled the absorption spectrum of the fully reduced FAD but not that of the iron-sulfur cluster. And there was no activity of *Se*PhrB under the light beyond 550 nm where the iron-sulfur cluster still had absorption. Nevertheless, the quantum yields of the reaction would be unacceptably low (<0.01) if the absorption of the iron-sulfur cluster was taken into account in the calculation. Therefore, we concluded that the absorption of iron-sulfur cluster does not contribute to the photorepair process. An intriguing function of the iron-sulfur cluster had been proposed, which might participate the DNA-mediated charge transfer for DNA damage detection and long-range communication between DNA repair and processing enzymes (53). The functions of the iron-sulfur cluster in FeS-BCPs warranted further investigation.

**Figure 6.**
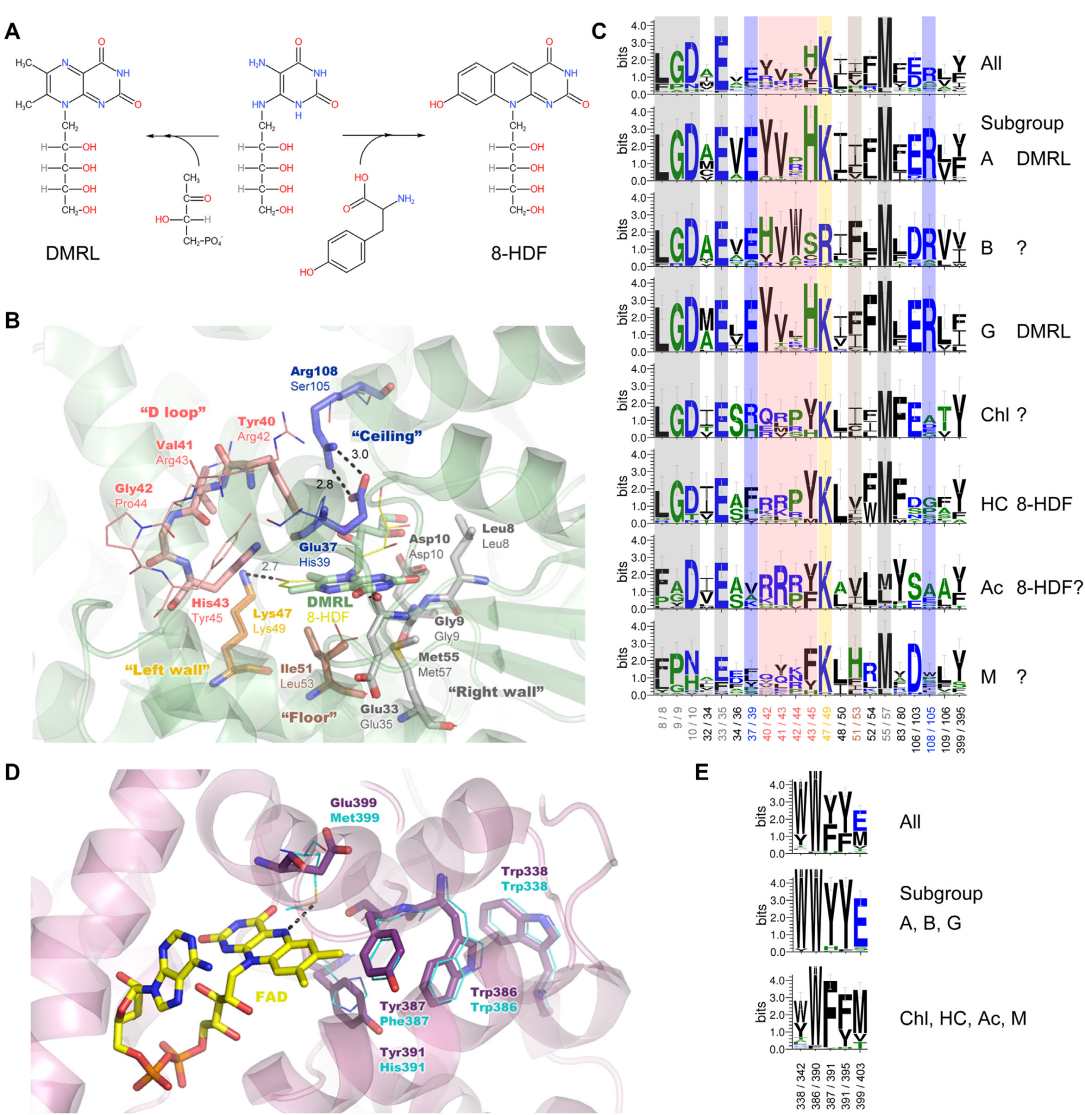
The antenna cofactor and FAD binding, and the putative electron transfer chain sites in *Se*PhrB and other prokaryotic 6–4 photolyases. (**A**) The formulas of DMRL and 8-HDF. The two cofactors have a common biosynthetic precursor (5-amino-6-(d-ribitylamino)uracil) and several similar features. (**B**) The structural details of the antenna cofactor binding pocket. The pale green cartoon shows the secondary structures of *Af*PhrB (PDB ID: 4DJA) (18) around DMRL (shown in the pale green stick representation). And the residues of *Af*PhrB interacting with DMRL are shown in the stick representations with different colors. The residues located at the ‘right wall’ of the binding pocket that interacting the ribityl moiety or the pyrimidine ring of the chromophore moiety of DMRL are shown in grey. The conserved lysine (Lys47) located at the ‘left wall’ is shown in orange. The apolar residue (Ile51) at the ‘floor’ is shown in brown. The residues in the ‘D loop’ at the top-left conner are shown in salmon. And two residues (Glu37 and Arg108) at the ‘ceiling’ that forming a salt bridge above the pyrazine ring of DMRL are shown in blue. The positions of the homologous residues in *Se*PhrB predicted by AlphaFold2 (33) are shown in line representations with corresponding colors used in *Af*PhrB. The position of the 8-HDF cofactor could not be predicted, which is temporally placed in the homologous position of DMRL in the yellow line representation. (**C**) The conservation of the antenna cofactor binding sites of all prokaryotic 6–4 photolyases and of those in different subgroups. The conserved residues at the ‘right wall’ are shown in grey. The possible salt bridge forming sites at the ‘ceiling’ are shown in blue. Those in the D loop are shown in salmon. The conserved lysine/arginine at the ‘left wall’ are shown in orange. And the residues at the ‘floor’ are shown in brown. The possible antenna cofactor types are listed following the subgroup names. (**D**) The structural details of the FAD binding pocket and the electron transfer chain. The pink cartoon shows the secondary structures of *Rs*CryB (PDB ID: 3ZXS) (19) around FAD (shown in the yellow stick representation). And the residue of *Rs*CryB located near the N5 position of FAD (Glu399) and those in the electron transfer chain are shown in the purple stick representations. The predicted positions of the homologous residues in *Se*PhrB are shown in cyan line representations. (**E**) The conservation of the sites in the putative electron transfer chain and that near the N5 position of FAD of all prokaryotic 6–4 photolyases; of the members in subgroups A, B, and G; and of those in the rest subgroups.

All previously described FeS-BCPs contain a DMRL cofactor as the antenna cofactor (18,19,21,22). Here, we demonstrated that *Se*PhrB does not possess a DMRL, but an 8-HDF cofactor as its antenna cofactor. The 8-HDF cofactor is a precursor of F420, which functions analogously to NAD as a two-electron, hydride-transfer coenzyme in a number of archaea and actinomycetes (45,54). Many photolyases also utilize 8-HDF as their antenna cofactor (7,26,27,39–43). The DMRL cofactor is an intermediate in the last step of riboflavin biosynthesis (55). These two cofactors have a common biosynthetic precursor (5-amino-6-(d-ribitylamino)uracil) (55–57) (Figure 6A). Both of them contain a ribityl moiety, but the chromophore moiety of DMRL is bicyclic and that of 8-HDF is tricyclic (Figure 6A). The utilization of 8-HDF as the antenna cofactor has several advantages over DMRL. The 8-HDF cofactor has higher extinction coefficient and fluorescence intensity than DMRL. Nevertheless, the absorption spectrum of 8-HDF gives a better overlap with the solar radiation spectrum at the earth surface (7). Therefore, the light absorption and energy transferring by 8-HDF is more efficient. However, many organisms could not synthesize 8-HDF. Therefore, the utilization of DMRL as the antenna cofactor of many FeS-BCPs might be an adaptation for the lack of the 8-HDF biosynthesis.

Due to the lack of crystal structure of *Se*PhrB, the exact 8-HDF binding sites of the protein is currently unclear. But considering the structural similarities of DMRL and 8-HDF, we speculated that 8-HDF should bind to *Se*PhrB in the homologous positions where DMRL binds to *Af*PhrB. Basing on the crystal structure of *Af*PhrB (PDB ID: 4DJA) (18), twenty-one sites were identified to participated the DMRL binding (Figure 1B, marked with circles). There are six bind sites that are most conserved in all FeS-BCPs (Leu8/8, Gly9/9, Asp10/10, Glu33/35, Lys47/49 and Met55/57 in *Af*PhrB/*Se*PhrB, Figure 6B and C). All these sites except for Lys47 in *Af*PhrB are involved in forming ‘right wall’ of the binding pocket (Figure 6B, grey sticks) that interacting with the ribityl moiety and the pyrimidine ring of the chromophore moiety of DMRL. The homologous residues in *Se*PhrB might also interact with the corresponding moieties of 8-HDF. Lys47 is located at ‘left wall’ of the binding pocket (Figure 6B, orange sticks), where a similar basic residue Arg51 was observed in *Se*PhrA, and Arg51 in *Xenopus laevis* eukaryotic 6–4 photolyase (*Xl*64), which is crucial for the binding of 8-HDF by forming a salt bridge to the 8-oxy group of the cofactor (15,27,43). The role of Lys47 in *Af*PhrB is not understood. Its distance to 6-methyl of DMRL is 4.9 Å. However, if an 8-HDF cofactor occupied the same position of DMRL, the distance of Lys47/49 to the 8-oxy group of 8-HDF would be 2.7 Å, a salt bridge might form between them as well in this situation (Figure 6B). Therefore, Lys47 in *Af*PhrB is likely to be a remnant of an ancient 8-HDF binding site. Ile51 in *Af*PhrB (Leu53 in *Se*PhrB) is located at the ‘floor’ of the binding pocket (Figure 6B, brown sticks). Similar apolar residues are found in *Se*PhrA (Leu55) and in *Xl*64 (Leu55) (27,43). A small loop of *Af*PhrB involving Tyr40, Val41, Gly42 and His43 is located at the top-left conner of the binding pocket, near the 7-methyl of DMRL (Figure 6B, salmon sticks). The steric hindrance effect of His43 was considered as the main reason for the binding of the bicyclic DMRL but not a tricyclic cofactor (18). Tyr40 supports His43 in forming the steric hindrance. In contrast, a homologous loop of *Se*PhrB is constituted by Arg42, Arg43, Pro44 and Tyr45. Although Tyr45 in *Se*PhrB is also bulky as His43 in *Af*PhrB, the Arg42 residue in *Se*PhrB may not push Tyr45 too close to the cofactor, therefore allowing a tricyclic cofactor to bind (Figure 6B, salmon lines). We named this loop ‘D loop’, because it may be crucial for determination of the kind of the binding cofactor. Another feature in *Af*PhrB that may affect the cofactor selection is a salt bridge forming between Glu37 and Arg108 that located at the ‘ceiling’ of the binding pocket, above the pyrazine ring of the chromophore moiety of DMRL (Figure 6, black dashes and blue sticks). The electrostatic effect of the salt bridge may facilitate the binding of DMRL (or flavin) with the polar pyrazine ring, but not 8-HDF with a hydrophobic pyridine ring in the middle of the chromophore moiety. In *Se*PhrB, the homologous positions of Glu37 and Arg108 in *Af*PhrB are replaced by His39 and Ser105. No salt bridge can form between these residues, which may make the binding of 8-HDF to be possible. To be noted, in other 8-HDF binding photolyases, the homologous residues of Glu37/His39 in *Af*PhrB/*Se*PhrB are generally apolar residues (Ile41 in *Se*PhrA and Phe41 in *Xl*64), which were proved to be important for 8-HDF binding (15,27,43).

The conservation of the putative antenna cofactor binding sites was analyzed for different prokaryotic 6–4 photolyase subgroups (Figure 6C). The residues in the D loop are highly conserved as ‘Y-V-X-H’ in the subgroups A and G. And the possible salt bridge forming residues at the ‘ceiling’ homologous to Glu37 and Arg108 are also conserved in these two subgroups. Therefore, it is highly possible that most members in the subgroups A and G bind DMRL. In the subgroups B, the residues in the D loop are conserved as ‘H-V-W-S’. Considering that serine is not a bulky residue, the members in the subgroups B may bind a tricyclic antenna cofactor. However, the possible salt bridge forming residues at the ‘ceiling’ are also conserved in the subgroups B as those in the subgroups A and G, which may be not favorable for the 8-HDF binding. For the subgroups Chl, HC, and Ac, the residues in the D loop are conserved as R/Q-R-P/R-Y; and the possible salt bridge forming residues at the ‘ceiling’ are less well conserved. This evidence suggests that the members in these three subgroups may contain 8-HDF as the antenna cofactor. However, although there is a report that some species of the phylum *Chloroflexi* are able to synthesize 8-HDF and F420 (57), the three *Chloroflexi* hosts (*Roseiflexus castenholzii, Chloroflexus aurantiacus, Chloroflexus aggregans*) and the *Chlorobi* host (*Chlorobium* sp.) of the members in the subgroup Chl of this analysis do not contain FO synthase genes. The actual antenna cofactors of the members in the subgroups Chl and Ac are to be determined. For the subgroup M, the conservation pattern is much complex, implying the antenna cofactors of this subgroup members are diverse that need extensive investigation.

Most photolyases and cryptochromes could be photoreduced in the presence of external electron donors. During photoreduction, an electrons transfers from a donor to FAD in the fully oxidized or radical state via a chain involving several conserved tryptophan/tyrosine residues. In the absence of external donors, back electron transfer occurred and the FAD cofactor would quickly return to its original redox state (46,47). However, it was observed that the photoreduction of *Se*PhrB did not need an external electron donor. And the oxidation of photoreduced *Se*PhrB was extremely slow compared with other photolyases. It was proposed that the electron transfer chain of a FeS-BCP contain two tryptophan and two tyrosine residues (Trp338, Trp386, Tyr387, and Tyr391 in *Rs*CryB, Figure 6D) (19). But based on the modelled structure of SePhrB, it was found that there is no tryptophan or tyrosine within 4 Å to the isoalloxazine ring of FAD. The putative electron transfer chain of *Se*PhrB according to that proposed in *Rs*CryB is interrupted that the two tyrosine residues is replaced with a phenylalanine and a histidine (Phe387 and His391, Figure 6D). Nevertheless, the photoreduction rate of *Se*PhrB was still faster than *Af*PhrB that contains the intact electron transfer chain (Trp342, Trp390, Tyr391 and Tyr395 in *Af*PhrB). The possibility that electrons come from iron-sulfur cluster to FAD in *Se*PhrB is unlikely, because their distance is ∼16.8 Å, and no conceivable electron transfer pathway is observed between them. Inspection of the sequences and structures revealed a unique residue located near the N5 position of FAD (Glu399/403 in *Rs*CryB/*Af*PhrB). It is replaced by Met399 in *Se*PhrB at the homologous position (Figure 6D). An interesting conservation pattern was observed that 89.8% (53/59) members in the subgroups A, B and G (but none of the members in the subgroups Chl, HC, Ac, and M) have the intact putative electron transfer chain. On the other hand, 91.5% (54/59) members in the subgroups A, B, and G contain a glutamic acid near the N5 position; and 74% (37/50) members in the subgroups Chl, HC, Ac and M contain a methionine at the homologous position (Figure 6E). Methionine is also a redox active residue, which may provide electrons in some conditions (58,59). Nevertheless, it was reported that in a mutant (C57M) of *C. reinhardtii* phototropin LOV1 domain, the methionine located at near the N5 position of the FMN cofactor could form a covalent linkage with FMN to produce a N5 adduct upon blue light illumination. The absorption properties of the N5 adduct resemble reduced flavin. It slowly converted into a radical state adduct which was stable for several months under aerobic conditions (60). It is currently under investigation that whether the similar reactions take place in *Se*PhrB upon illumination, and whether the reactions have relationship to its repair function. To fully elucidate the distinctive properties of *Se*PhrB, further mutagenesis and crystallography experiments are planned. Considering that *Se*PhrB has high repair activity for 6–4 photoproducts, is readily photoreduced to the active form without the need of external reductants, and has extreme stability in the active form against oxidation, this enzyme is a good candidate for topical application together with another CPD photolyase to reverse DNA damage in skin cells, and to prevent the development of many skin diseases (61,62).

## DATA AVAILABILITY

The predicted structure of *Se*PhrB has been deposited to Model Archive with the accession code ma-r6fuc.

## Supplementary Material

gkac416_Supplemental_File
