# Supplementary material for: Identification and characterization of a prokaryotic 6-4 photolyase from Synechococcus elongatus with a deazariboflavin antenna chromophore
Source: Nucleic Acids Res. 2022 May 27;50(10):5757–71. doi: 10.1093/nar/gkac416 (PMC9178010; doi:10.1093/nar/gkac416)
Supplement: gkac416_Supplemental_File [file gkac416_supplemental_file.pdf]

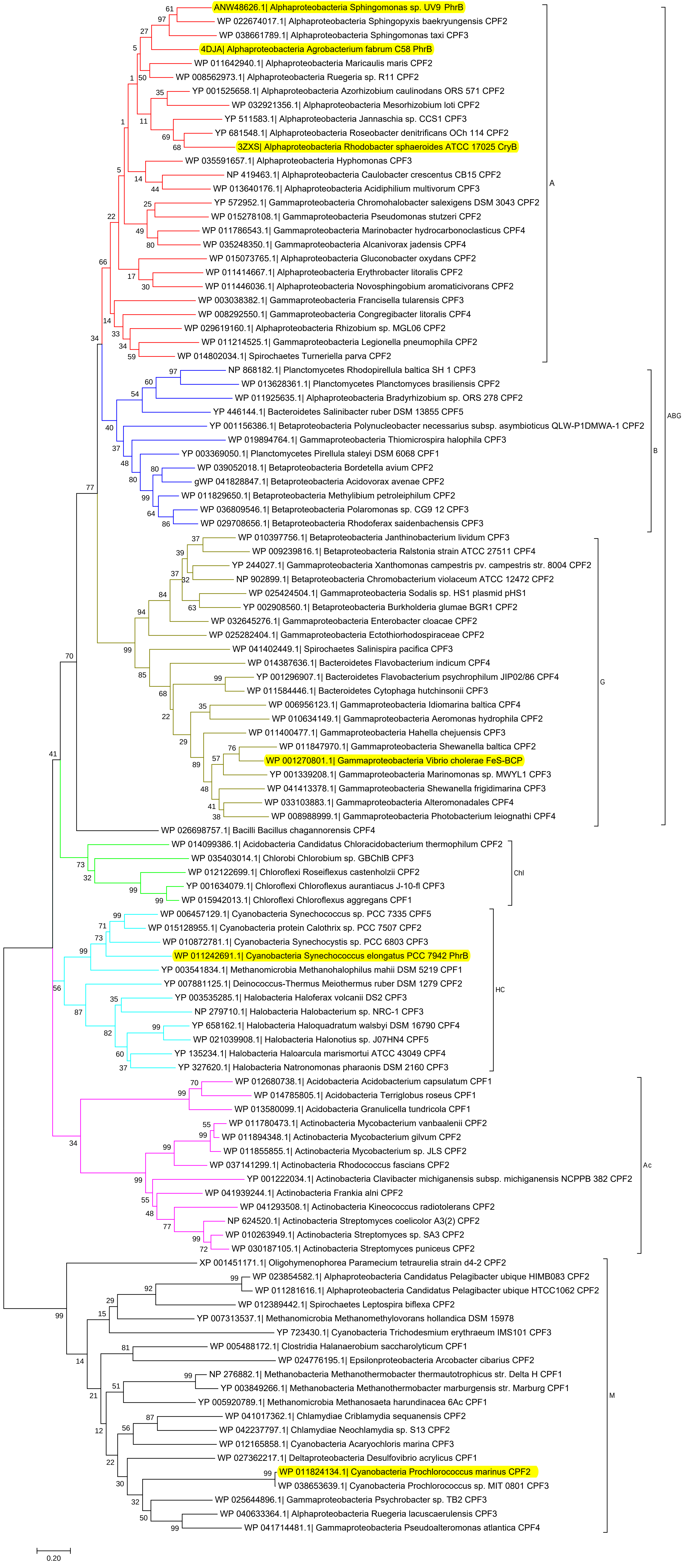

ANW48626.1| Alphaproteobacteria Sphingomonas sp. UV9 PhrB
WP 022674017.1| Alphaproteobacteria Sphingopyxis baekryungensis CPF2
WP 038661789.1| Alphaproteobacteria Sphingomonas taxi CPF3
4DJA| Alphaproteobacteria Agrobacterium fabrum C58 PhrB
WP 011642940.1| Alphaproteobacteria Maricaulis maris CPF2
WP 008562973.1| Alphaproteobacteria Ruegeria sp. R11 CPF2
YP 001525658.1| Alphaproteobacteria Azorhizobium caulinodans ORS 571 CPF2
WP 032921356.1| Alphaproteobacteria Mesorhizobium loti CPF2
YP 511583.1| Alphaproteobacteria Jannaschia sp. CCS1 CPF3
YP 681548.1| Alphaproteobacteria Roseobacter denitrificans OCh 114 CPF2
3ZXS| Alphaproteobacteria Rhodobacter sphaeroides ATCC 17025 CryB
WP 035591657.1| Alphaproteobacteria Hyphomonas CPF3
NP 419463.1| Alphaproteobacteria Caulobacter crescentus CB15 CPF2
WP 013640176.1| Alphaproteobacteria Acidiphilium multivorum CPF3
YP 572952.1| Gammaproteobacteria Chromohalobacter salexigens DSM 3043 CPF2
WP 015278108.1| Gammaproteobacteria Pseudomonas stutzeri CPF2
WP 011786543.1| Gammaproteobacteria Marinobacter hydrocarbonoclasticus CPF4
WP 035248350.1| Gammaproteobacteria Alcanivorax jadensis CPF4
WP 015073765.1| Alphaproteobacteria Gluconobacter oxydans CPF2
WP 011414667.1| Alphaproteobacteria Erythrobacter litoralis CPF2
WP 011446036.1| Alphaproteobacteria Novosphingobium aromaticivorans CPF2
WP 003038382.1| Gammaproteobacteria Francisella tularensis CPF3
WP 008292550.1| Gammaproteobacteria Congregibacter litoralis CPF4
WP 029619160.1| Alphaproteobacteria Rhizobium sp. MGL06 CPF2
WP 011214525.1| Gammaproteobacteria Legionella pneumophila CPF2
WP 014802034.1| Spirochaetes Turneriella parva CPF2
A
NP 868182.1| Planctomycetes Rhodopirellula baltica SH 1 CPF3
WP 013628361.1| Planctomycetes Planctomyces brasiliensis CPF2
WP 011925635.1| Alphaproteobacteria Bradyrhizobium sp. ORS 278 CPF2
YP 446144.1| Bacteroidetes Salinibacter ruber DSM 13855 CPF5
YP 001156386.1| Betaproteobacteria Polynucleobacter necessarius subsp. asymbioticus QLW-P1DMWA-1 CPF2
WP 019894764.1| Gammaproteobacteria Thiomicrospira halophila CPF3
YP 003369050.1| Planctomycetes Pirellula staleyi DSM 6068 CPF1
WP 039052018.1| Betaproteobacteria Bordetella avium CPF2
gWP 041828847.1| Betaproteobacteria Acidovorax avenae CPF2
WP 011829650.1| Betaproteobacteria Methylibium petroleiphilum CPF2
WP 036809546.1| Betaproteobacteria Polaromonas sp. CG9 12 CPF3
WP 029708656.1| Betaproteobacteria Rhodoferax saidenbachensis CPF3
B
ABG
WP 010397756.1| Betaproteobacteria Janthinobacterium lividum CPF3
WP 009239816.1| Betaproteobacteria Ralstonia strain ATCC 27511 CPF4
YP 244027.1| Gammaproteobacteria Xanthomonas campestris pv. campestris str. 8004 CPF2
NP 902899.1| Betaproteobacteria Chromobacterium violaceum ATCC 12472 CPF2
WP 025424504.1| Gammaproteobacteria Sodalis sp. HS1 plasmid pHS1
YP 002908560.1| Betaproteobacteria Burkholderia glumae BGR1 CPF2
WP 032645276.1| Gammaproteobacteria Enterobacter cloacae CPF2
WP 025282404.1| Gammaproteobacteria Ectothiorhodospiraceae CPF2
WP 041402449.1| Spirochaetes Salinispira pacifica CPF3
WP 014387636.1| Bacteroidetes Flavobacterium indicum CPF4
YP 001296907.1| Bacteroidetes Flavobacterium psychrophilum JIP02/86 CPF4
WP 011584446.1| Bacteroidetes Cytophaga hutchinsonii CPF3
WP 006956123.1| Gammaproteobacteria Idiomarina baltica CPF4
WP 010634149.1| Gammaproteobacteria Aeromonas hydrophila CPF2
WP 011400477.1| Gammaproteobacteria Hahella chejuensis CPF3
WP 011847970.1| Gammaproteobacteria Shewanella baltica CPF2
WP 001270801.1| Gammaproteobacteria Vibrio cholerae FeS-BCP
YP 001339208.1| Gammaproteobacteria Marinomonas sp. MWYL1 CPF3
WP 041413378.1| Gammaproteobacteria Shewanella frigidimarina CPF3
WP 033103883.1| Gammaproteobacteria Alteromonadales CPF4
WP 008988999.1| Gammaproteobacteria Photobacterium leiognathi CPF4
G
WP 026698757.1| Bacilli Bacillus chagannorensis CPF4
WP 014099386.1| Acidobacteria Candidatus Chloracidobacterium thermophilum CPF2
WP 035403014.1| Chlorobi Chlorobium sp. GBChlB CPF3
WP 012122699.1| Chloroflexi Roseiflexus castenholzii CPF2
YP 001634079.1| Chloroflexi Chloroflexus aurantiacus J-10-fl CPF3
WP 015942013.1| Chloroflexi Chloroflexus aggregans CPF1
Chl
WP 006457129.1| Cyanobacteria Synechococcus sp. PCC 7335 CPF5
WP 015128955.1| Cyanobacteria protein Calothrix sp. PCC 7507 CPF2
WP 010872781.1| Cyanobacteria Synechocystis sp. PCC 6803 CPF3
WP 011242691.1| Cyanobacteria Synechococcus elongatus PCC 7942 PhrB
YP 003541834.1| Methanomicrobia Methanohalophilus mahii DSM 5219 CPF1
YP 007881125.1| Deinococcus-Thermus Meiothermus ruber DSM 1279 CPF2
YP 003535285.1| Halobacteria Haloferax volcanii DS2 CPF3
NP 279710.1| Halobacteria Halobacterium sp. NRC-1 CPF3
YP 658162.1| Halobacteria Haloquadratum walsbyi DSM 16790 CPF4
WP 021039908.1| Halobacteria Halonotius sp. J07HN4 CPF5
YP 135234.1| Halobacteria Haloarcula marismortui ATCC 43049 CPF4
YP 327620.1| Halobacteria Natronomonas pharaonis DSM 2160 CPF3
HC
WP 012680738.1| Acidobacteria Acidobacterium capsulatum CPF1
WP 014785805.1| Acidobacteria Terriglobus roseus CPF1
WP 013580099.1| Acidobacteria Granulicella tundricola CPF1
WP 011780473.1| Actinobacteria Mycobacterium vanbaalenii CPF2
WP 011894348.1| Actinobacteria Mycobacterium gilvum CPF2
WP 011855855.1| Actinobacteria Mycobacterium sp. JLS CPF2
WP 037141299.1| Actinobacteria Rhodococcus fascians CPF2
YP 001222034.1| Actinobacteria Clavibacter michiganensis subsp. michiganensis NCPPB 382 CPF2
WP 041939244.1| Actinobacteria Frankia alni CPF2
WP 041293508.1| Actinobacteria Kineococcus radiotolerans CPF2
NP 624520.1| Actinobacteria Streptomyces coelicolor A3(2) CPF2
WP 010263949.1| Actinobacteria Streptomyces sp. SA3 CPF2
WP 030187105.1| Actinobacteria Streptomyces puniceus CPF2
Ac
XP 001451171.1| Oligohymenophorea Paramecium tetraurelia strain d4-2 CPF2
WP 023854582.1| Alphaproteobacteria Candidatus Pelagibacter ubique HIMB083 CPF2
WP 011281616.1| Alphaproteobacteria Candidatus Pelagibacter ubique HTCC1062 CPF2
WP 012389442.1| Spirochaetes Leptospira biflexa CPF2
YP 007313537.1| Methanomicrobia Methanomethylovorans hollandica DSM 15978
YP 723430.1| Cyanobacteria Trichodesmium erythraeum IMS101 CPF3
WP 005488172.1| Clostridia Halanaerobium saccharolyticum CPF1
WP 024776195.1| Epsilonproteobacteria Arcobacter cibarius CPF2
NP 276882.1| Methanobacteria Methanothermobacter thermautotrophicus str. Delta H CPF1
YP 003849266.1| Methanobacteria Methanothermobacter marburgensis str. Marburg CPF1
YP 005920789.1| Methanomicrobia Methanosaeta harundinacea 6Ac CPF1
WP 041017362.1| Chlamydiae Criblamydia sequanensis CPF2
WP 042237797.1| Chlamydiae Neochlamydia sp. S13 CPF2
WP 012165858.1| Cyanobacteria Acaryochloris marina CPF3
WP 027362217.1| Deltaproteobacteria Desulfovibrio acrylicus CPF1
WP 011824134.1| Cyanobacteria Prochlorococcus marinus CPF2
WP 038653639.1| Cyanobacteria Prochlorococcus sp. MIT 0801 CPF3
WP 025644896.1| Gammaproteobacteria Psychrobacter sp. TB2 CPF3
WP 040633364.1| Alphaproteobacteria Ruegeria lacuscaerulensis CPF3
WP 041714481.1| Gammaproteobacteria Pseudoalteromonas atlantica CPF4
M
0.20

Figure S1 Phylogenetic tree of prokaryotic 6-4 photolyases. The NCBI RefSeq ID, the name of the species of each sequence are shown. The evolutionary history was inferred by using the maximum likelihood method based on the Le_Gascuel_2008 model and 500 bootstrap iterations. A discrete Gamma distribution was used to model evolutionary rate differences among sites (5 categories (+G, parameter = 1.1274)). The rate variation model allowed for some sites to be evolutionarily invariable ([+I], 5.09% sites). The analysis involved 110 amino acid sequences. All positions containing gaps and missing data were eliminated. There were 265 positions in the final dataset. Numbers at nodes represent the bootstrap support percentage values. The prokaryotic 6-4 photolyases are divided into seven subgroups (A, B, G, Chl, HC, Ac, and M). *Bacillus chagannorensis* FeS-BCP does not belong to any subgroups. The positions of the previously characterized members and *Se*PhrB are highlighted with yellow boxes.

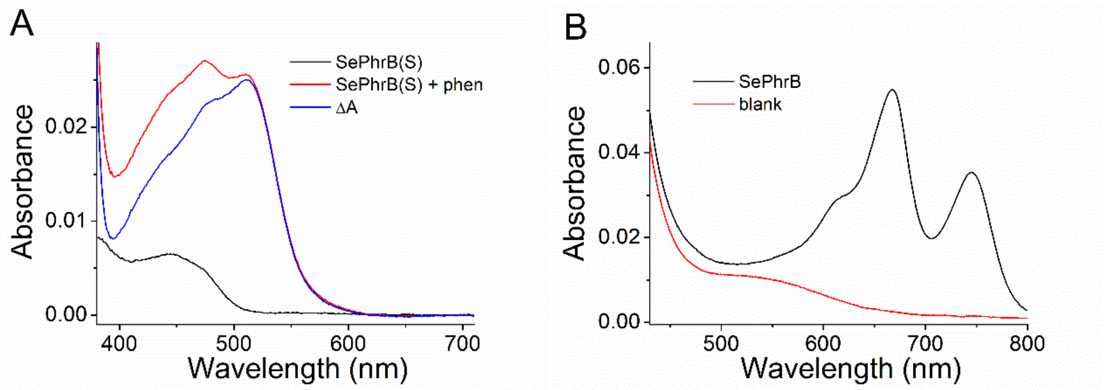

Figure S2 Determination of iron (A) and sulfur (B) contents in *Se*PhrB. (A) The protein sample of 500 µl was mixed with 25 µl concentrated HCl, and heated for 10 min to 80°C. The spectrum of the supernatant (*Se*PhrB(S)) was shown in black line. Then the supernatant was mixed with 250 µl of 0.2 M potassium biphthalate and 500 µl of 0.3% (w/v) 1,10-phenanthroline (phen). The spectrum of the solution (*Se*PhrB (S) + phen) is shown in red line. The blue line (ΔA) is the difference spectrum of *Se*PhrB (S) + phen and *Se*PhrB(S). The absorbance at 511 nm is proportional to the iron content of the sample. The spectra were corrected for dilution. (B) The protein sample of 400 µl was mixed with 200 µl of Zn acetate (1% w/v) and 10 µl of NaOH (12% w/v). After incubation, 100 µl of 1% N,N'-dimethyl-p-phenylenediamine (DMPD) and 20 µl of 23 mM $FeCl_3$ was added. The spectrum of the supernatant (*Se*PhrB) is shown in black line. The absorbance at 745 nm is proportional to the sulfur content of the sample. The red line is a result of a blank determination.

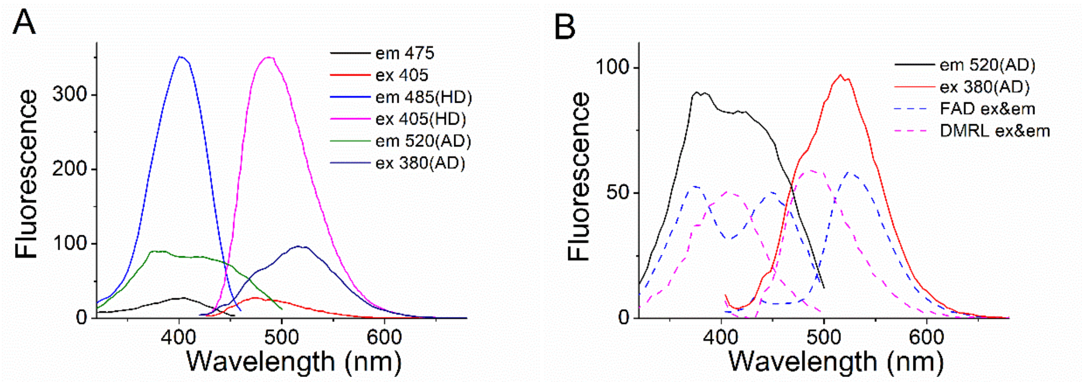

Figure S3 The fluorescence spectra of *Af*PhrB. (A) The emission and excitation spectra of native *Af*PhrB, and the supernatants of heat denatured (HD) or acid denatured (AD) *Af*PhrB. (B) The magnified fluorescence spectra of the supernatants of acid-denatured (AD) *Af*PhrB, which could be roughly divided into DMRL and FAD components that are shown in dash lines. The protein concentrations used in the analyses were ~2 μM.

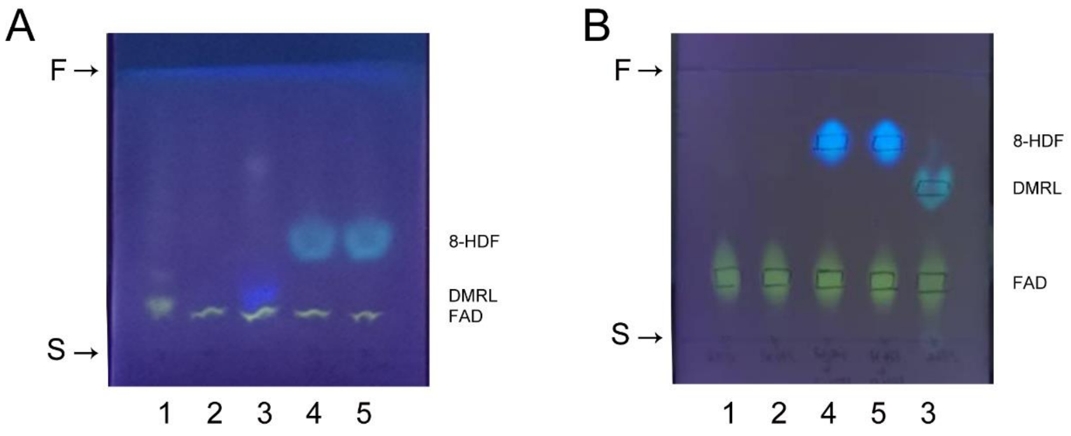

Figure S4 The thin-layer chromatography of authentic FAD (lane 1), and the supernatants of heat denatured *Se*PhrB that expressed in *E. coli* (*Se*PhrB[*Ec*], lane 2), *Af*PhrB (lane 3), *Se*PhrA co-expressed with *Sc*FbiC (*Se*PhrA[*Sc*FbiC], lane 4), and *Se*PhrB co-expressed with *Sc*FbiC (*Se*PhrB[*Sc*FbiC], lane 5). The start and front positions (S and F) are indicated by arrows. The solvent systems were: (A) *n*-butanol/ethanol/water, 10:3:7; (B) acetonitrile/water/formic acid (88%), 40:10:5.

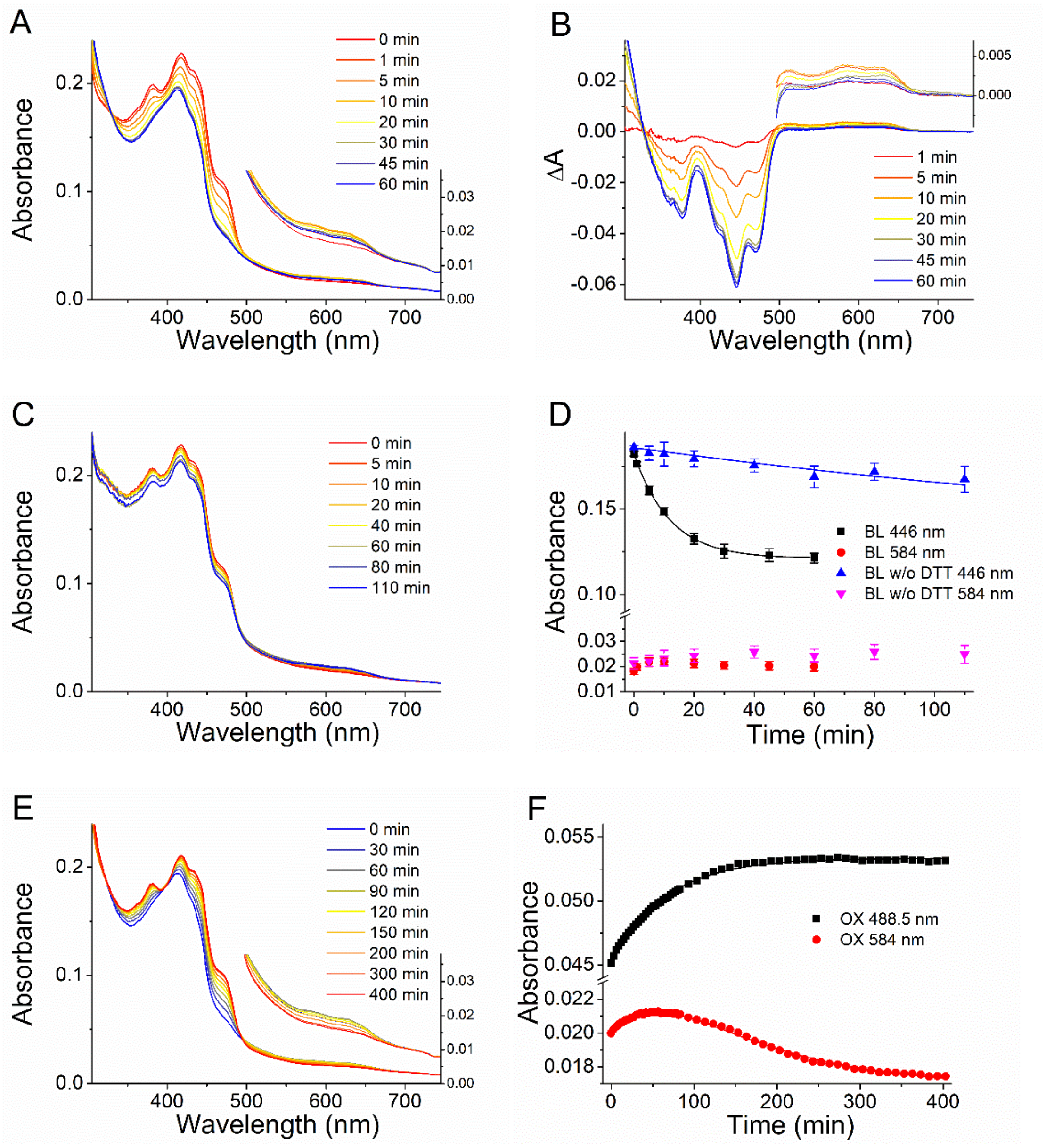

Figure S5 Photoreduction and oxidation of *Af*PhrB. (A) The *Af*PhrB sample (5.2 μM) was illuminated under blue light (λ_{max} = 440 nm, irradiance of ~190 W m^{-2}) with 10 mM DTT. The absorption spectra were recorded at indicated intervals. The inset shows the magnified spectra in the 500-750 nm range. (B) The difference spectra of illuminated and non-illuminated *Af*PhrB calculated from the data shown in (A). The inset shows the magnified spectra in the 500-750 nm range. (C) The *Af*PhrB sample (5.2 μM) was illuminated under the blue light without DTT. (D) The photoreduction kinetics of *Af*PhrB under the blue light with 10 mM and without DTT depicted using the absorbance changes at the peak of fully oxidized FAD (446 nm) and those at the peak of neutral radical FAD (584 nm) in *Af*PhrB. Data points represent means ± SD (n = 3). The absorbance changes at 584 nm were relatively small that could be negligible. And the absorbance changes at 446 nm were fitted with a mono-exponential function to obtain pseudo photoreduction rate constants (k_{pr}) to be 1.4 ± 0.3 × 10^{-3} sec^{-1} and 6.2 ± 1.3 × 10^{-5} sec^{-1} for the

*Af*PhrB samples with and without DTT, respectively. The R^2 values of the fittings were 0.999 and 0.941, respectively. (E) The photoreduced *Af*PhrB sample was oxidized in the dark under aerobic conditions at 18 ± 0.5°C. The absorption spectra were recorded at indicated intervals. (F) The oxidation kinetics of *Af*PhrB depicted using the absorbance change at the isosbestic wavelength of neutral radical and fully oxidized FAD (488.5 nm) and that at the peak of neutral radical FAD (584 nm) in *Af*PhrB. The data points were analyzed using a sequential reaction model (from the fully reduced state to the neutral radical state, and from the neutral radical state to the fully oxidized state). The oxidation rate constant from the fully reduced state to the neutral radical state (k_{ox1}) and that from the neutral radical state to the fully oxidized state (k_{ox2}) were calculated to be $2.6 \pm 0.3 \times 10^{-4}$ sec^{-1} and $1.5 \pm 0.6 \times 10^{-4}$ sec^{-1}, respectively.

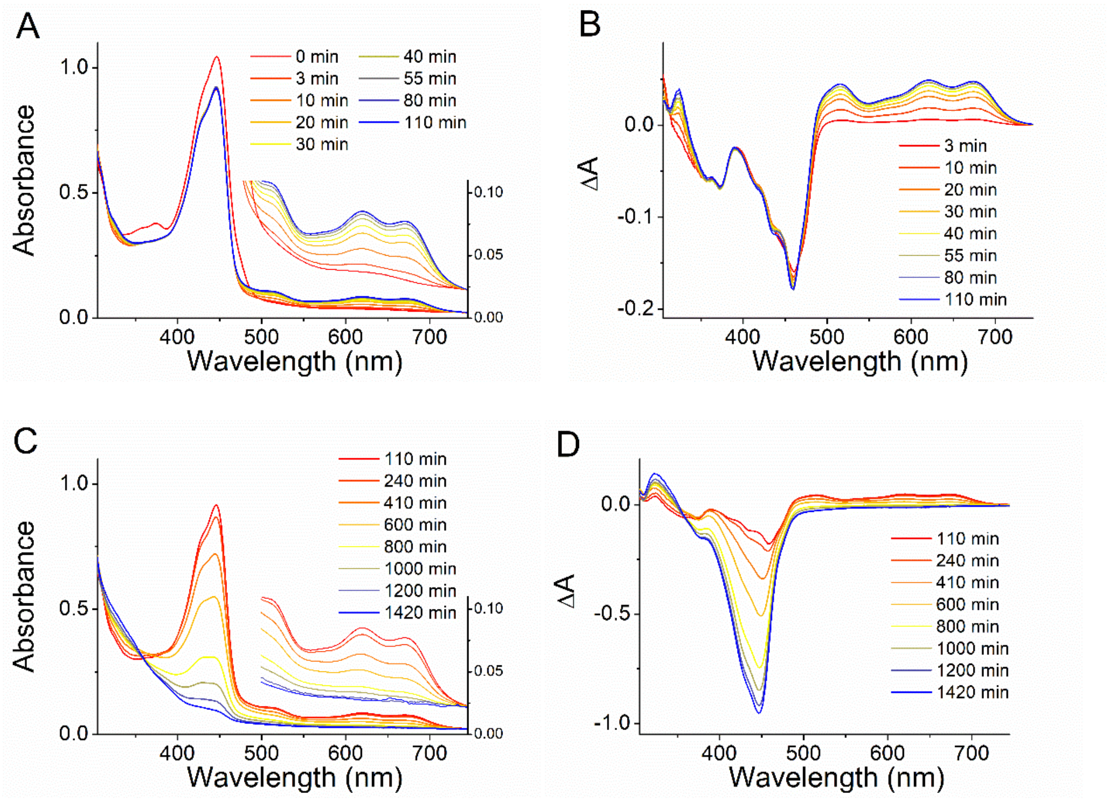

Figure S6 Photoreduction and Photo-oxidation of *Se*PhrB[8-HDF]. The *Se*PhrB[8-HDF] sample (12.9 μM) was illuminated under blue light (λ_{max} = 440 nm, irradiance of ~190 W m^{-2}) without DTT. The absorption spectra were recorded during illumination for 0-110 min (A) and 110-1420 min (C) at indicated intervals. The insets show the magnified spectra in the 500-750 nm range. (B) and (D) The difference spectra of illuminated and non-illuminated *Se*PhrB[8-HDF] calculated from the data shown in (A) and (C). The FAD cofactor in the *Se*PhrB[8-HDF] sample was first photoreduced from the fully oxidized state to fully reduced state (0-3 min), and then photo-oxidized to the neutral radical state (3-110 min). The radical FAD was photoreduced again together with 8-HDF during prolonged illumination (110-1420 min), leaving a low absorption shoulder at 447 nm.
